# Comparing fixed-array and functionally-defined channel of interest approaches to infant functional near-infrared spectroscopy data

**DOI:** 10.1016/j.neuroimage.2022.119520

**Published:** 2022-07-25

**Authors:** Yiyu Liu, Fernando Sánchez Hernández, Fransisca Ting, Daniel C. Hyde

**Affiliations:** a University of Illinois at Urbana-Champaign, Department of Psychology, Champaign, United States; b University of Illinois at Urbana-Champaign, Neuroscience Program, Urbana, United States; c Boston University, Department of Psychological and Brain Sciences, Boston, United States

**Keywords:** Fixed-array analysis, Infant, fNIRS, Cognition, Temporal lobe, fCOI

## Abstract

Functional near-infrared spectroscopy (fNIRS) is increasingly used to study brain function in infants, but the development and standardization of analysis techniques for use with infant fNIRS data have not paced other technical advances. Here we quantify and compare the effects of different methods of analysis of infant fNIRS data on two independent fNIRS datasets involving 6–9-month-old infants and a third simulated infant fNIRS dataset. With each, we contrast results from a traditional, fixed-array analysis with several functional channel of interest (fCOI) analysis approaches. In addition, we tested the effects of varying the number and anatomical location of potential data channels to be included in the fCOI definition. Over three studies we find that fCOI approaches are more sensitive than fixed-array analyses, especially when channels of interests were defined within-subjects. Applying anatomical restriction and/or including multiple channels in the fCOI definition does not decrease and in some cases increases sensitivity of fCOI methods. Based on these results, we recommend that researchers consider employing fCOI approaches to the analysis of infant fNIRS data and provide some guidelines for choosing between particular fCOI approaches and settings for the study of infant brain function and development.

## 1. Introduction

Over the last decade there has been a dramatic increase in the application of functional near-infrared spectroscopy (fNIRS) to study brain function in infants and young children due to its unique advantages over other common neuroimaging methods for testing developmental populations such as electroencephalogram (EEG) and functional magnetic resonance imaging (fMRI; Aslin, 2012; Gervain et al., 2011; Lloyd-Fox et al., 2010; Minagawa-Kawai et al., 2008). For example, fNIRS data can be collected from infants in naturalistic settings such as sitting on a parent’s lap or in a highchair. In contrast, fMRI requires participants to lay in a scanner bore and remain still and, as such, cannot yet be widely or easily applied to awake populations of infants and very young children. FNIRS also has better spatial resolution compared to EEG (Gervain et al., 2011), another widely used infant brain measure. Whereas the cortical source of EEG is difficult to definitively determine, as the electrical activity measured by EEG propagates and sums across the scalp, physical constraints on light propagation and detection through underlying tissue reduce source localization estimates in NIRS data to a few centimeters around a given data channel’s (light source-detector pair) scalp placement (Cui et al., 2011; Luck, 2005; Minagawa-Kawai et al., 2008). The increase in use and application of fNIRS has been accompanied by advances in hardware, software, and head probes to meet data collection and data processing challenges (Aasted et al., 2015; Gervain et al., 2011; Goodwin et al., 2016; Huppert et al., 2009; Lloyd-Fox et al., 2010; Santosa et al., 2018; Ye et al., 2009; Yücel et al., 2017; Saikia et al., 2019; Zimmermann et al., 2019). However, parallel advances in data analysis are needed for fNIRS to become and remain a premier method for studying brain function in infants (Gervain et al., 2011; Powell et al., 2018; Gemignani and Gervain, 2021a, 2021b). Here we add to knowledge of infant fNIRS data analysis by directly comparing and contrasting outcomes using different methods of analysis. Specifically, we compare a commonly used fixed-array analysis approach with emerging alternatives that use functional brain data itself to define channels of interest for further analysis, heretofore the functional channel of interest (fCOI) approach, on three datasets. Furthermore, we also directly compare and quantify the effects of several important analytic choices within fCOI analyses on each dataset.

Like other neuroimaging modalities, fNIRS data is often collected from multiple channels and, correspondingly, multiple regions of the brain simultaneously (Singh and Dan, 2006). Since fNIRS systems are often limited in the number of measurement channels available, it is common for researchers to attempt to strategically target more specific brain regions. This is often done based on coarse anatomical information (Genovese et al., 2002; Logan and Rowe, 2004). For example, in the typical fNIRS experiment the researcher places the headgear (i.e., probe) on the participant relative to scalp landmarks that are thought to correspond to the brain region(s) of interest for the particular study (Gervain et al., 2011). Placement is guided by known correspondences between scalp landmarks and underlying brain regions established with MRI as well as multimodal MRI-NIRS in adults (Ferradal et al., 2014; Jurcak et al., 2007; Okamoto et al., 2004; Singh et al., 2005; Tsuzuki et al., 2007). From this work, certain scalp landmarks have been shown, on average, to correspond probabilistically to known brain regions. Inferences based on this work allow the researcher to focus recordings to channels with a greater likelihood of corresponding to the underlying brain regions of interest.

Many infant fNIRS data analysis approaches currently used assume that a given channel in a head probe is measuring from the same underlying brain region(s) across participants in the sample (see Powell et al., 2018 for a review). The most common of these approaches, at least historically in the published infant fNIRS literature, is what we refer to as the fixed-array approach (see Powell et al., 2018). In the traditional instantiation of a fixed-array approach, researchers aggregate data from different participants by channel and test for the effects of interest in this “channel-space ” (Ferreri et al., 2013; Gervain et al., 2008; Grossmann et al., 2008; Lloyd-Fox et al., 2009). Of course, as the number of channels analyzed increases so does the likelihood of a statistical false positive (Ranganathan et al., 2016). Even if recordings are only taken in more focused scalp regions or analyses are restricted to certain portions of the probe, multiple data channels are still likely available for analysis. In some published cases, especially in the past, fNIRS researchers have ignored the multiple comparisons issue in favor of identifying potential effects of interests. Others have adjusted the statistical threshold for significance using standard multiple testing techniques (Shaffer, 1995) such as Bonferroni correction or False Discovery Rate (FDR) to try to account for multiple comparisons but doing so may also be accompanied by a reduction in sensitivity to the effects of interest (Singh and Dan, 2006). Still, others have used more data-driven approaches to spatially (and temporally) reduce the data, identifying potential effects and then using statistical thresholds derived from random permutations of the data to determine which effects are less likely to have occurred by chance (e.g., Abboub et al., 2016; Edwards et al., 2016; Ferry et al., 2016; Fló et al., 2019; Mahmoudzadeh et al., 2013).

Whether or not additional methods are used to reduce data and/or control for multiple comparisons, a similar assumption guides these sorts of “fixed-array” or “channel-space” analyses common in the literature: that a given channel measures from the same brain region or regions across participants. Although fixed-array methods may be sufficient to infer average scalp-to-brain-correspondences and subsequently reduce the analysis to channels likely corresponding to brain regions of interest in adults (and older children), they are more limited in infant developmental populations for several reasons. First, there is less work establishing scalp-brain correspondences in infants of different ages compared to adults and standard average brain templates used for source localization modeling are not widely available in commonly used formats for many narrow yet developmentally important age ranges in infancy (Lloyd-Fox et al., 2014; Matsui et al., 2014, but see Almli et al., 2007; Sanchez et al., 2011). Second, individual difference factors that create variability in scalp-to-brain estimates such as head size or lack of systematicity of probe placement between individual participants are often exacerbated in infant populations compared to adults or older children (Gervain et al., 2011). Third, given the spatial resolution limits of fNIRS, a given data channel is more likely to record from multiple brain regions in infants compared to adults. All these factors compound to make inferences about localization less certain across infants and, as a result, less reliable as an information base for reducing recordings and subsequent analysis to the most relevant channels.

Emerging approaches relax the assumption of a fixed-array or “channel-space” analyses across participants. One such promising approach is to further develop participant-specific anatomical localization techniques, which may allow more direct access to the actual channel-brain correspondences in individual participants. In adults, localization can be further enhanced by obtaining additional information on individual head probe-scalp correspondences (e.g., 3-D probe digitization) in relation to a template brain or subject-specific MRI of the brain. Although these methods are more challenging in infants, there have been some promising recent advances on this front (Emberson et al., 2015, 2017; Jaffe-Dax et al., 2020).

A complementary method for data reduction common in the cognitive neurosciences is to constrain the analysis based on information in the functional response itself (Poldrack, 2007). The basic idea is that regions of interest, defined in voxels for fMRI or channels for fNIRS, can be determined by the functional response properties of the brain of individuals in addition to purely anatomical location. An extensive review, debate, and defense of the logic behind functional localizers is beyond the scope of this report, but the merits and limits have been extensively debated in the fMRI literature (Friston et al., 2006; Saxe et al., 2006). To summarize, since functional subdivisions in the brain do not always have clear anatomical distinctions and individuals have been shown to differ in regional functional organizations, using functional data to flexibly guide analyses can contribute novel information to brain localization as well as further constrain the focus of analyses.

Although localization or data reduction based on functional data itself has been widely used in the fMRI literature for decades, it has not yet been widely applied to fNIRS data. That being said, several recent reports show great promise of this method for overcoming some of the unique challenges of using fNIRS in infants. For example, a recent study directly comparing a fixed-array analysis and a fCOI analysis approach on the same dataset showed the fCOI approach to be a more sensitive method for identifying face- and scene-selective regions of posterior temporal lobe of the infant brain (Powell et al., 2018). Although not the purpose of another study from our group, we were able to fully replicate a previously observed pattern of results in a new (second) sample of participants with a different headgear configuration when a fCOI approach was applied (in contrast to the application of a fixed-array approach that only led to a partial replication, Hyde et al., 2018). We reasoned that this was because the fCOI approach was able to flexibly align and identify functional brain regions of interest across samples and differing head probes by relaxing the fixed-array analysis assumption that a given channel measures from the same brain region across contexts. These recent examples suggest that using a fCOI approach may provide additional sensitivity compared to approaches which assume a fixed-array (or fixed channel-space) across infant participants.

**Fig. 1.**
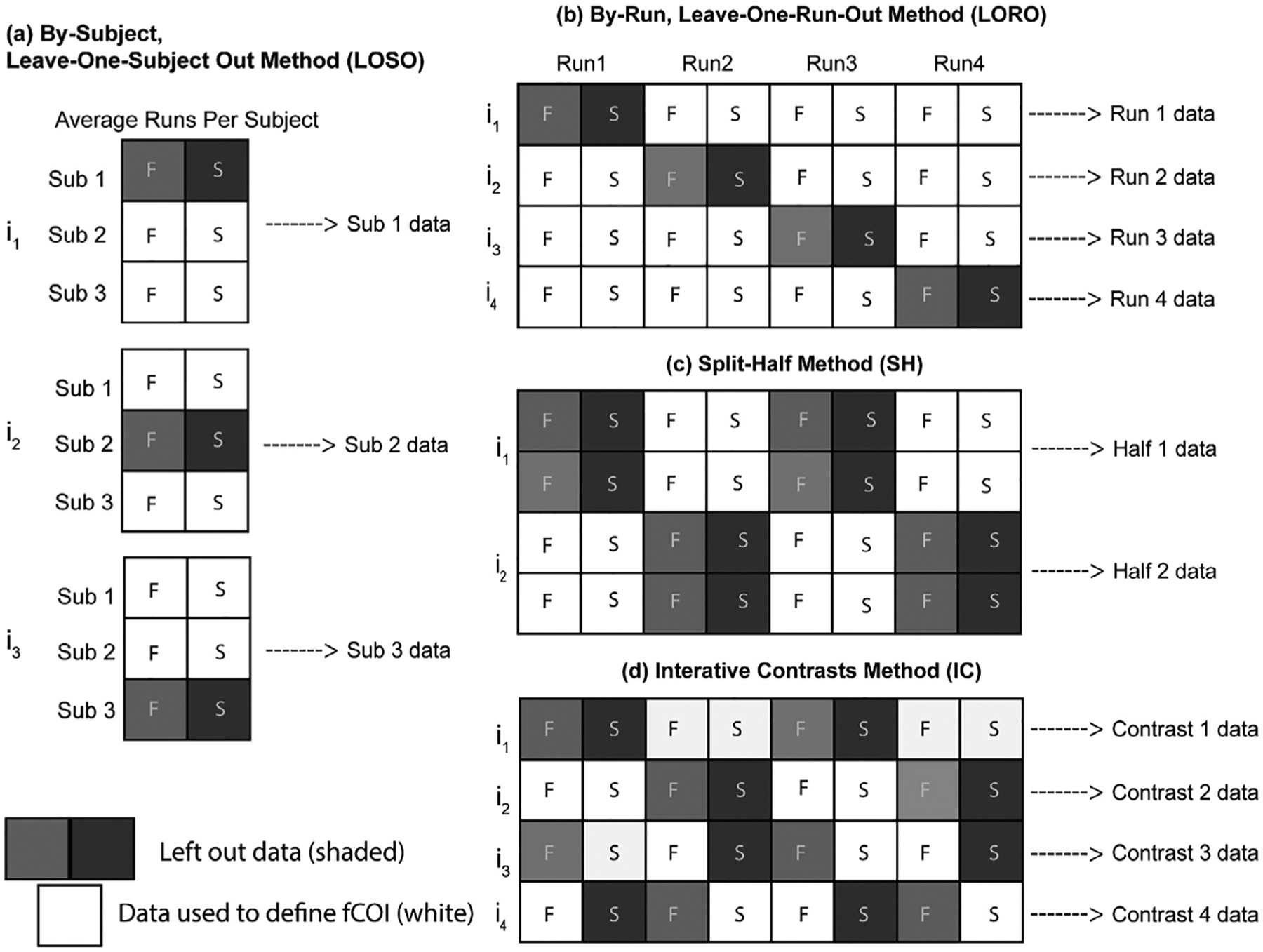
Schematic examples of different methods of sub-setting data for defining channels of interest independently of test data. The following is an example of study (similar to Study 1) involving multiple runs with each run including a face block (denoted F) and a scene block (denoted S). In this design, face and scene processing is contrasted to identify face-selective responses (face processing > scene processing). (a) The between-subject, leave-one subject out method example represents average response data for face processing and a scene processing control condition for three subjects. Over 3 iterations (i_1_,i_2_,i_3_), each subject’s data is held out (shaded boxes) and then a COI is defined based on functional data from the remaining subjects’ data (white boxes). (b) The within-subjects leave-one-run out method example represents four runs of data for a single example subject. Over four iterations (i_1_-i_4_), data from each run is held out (shaded boxes) and then a COI is defined based on the functional data from the remaining runs (white boxes). (c) The split-half method example represents four runs of data for a single example subject. Over two iterations (i_1_-i_2_), each half (odd or even) of the data is held out (shaded boxes) and then a COI is defined based on the functional data from the other half of the data (white boxes). (d) The iterative contrasts method example represents four runs of data for a single example subject. Over four iterations (i_1_-i_4_), each unique pairing of the odd and even halves of the face processing and scene processing runs is held out (shaded boxes) and a COI is defined based on the functional data from the remaining half of the data (white boxes).

Although fCOI analyses hold promise, there is not yet consensus on the best way to conduct them with infant fNIRS data. Previously published infant fNIRS studies report different analytic choices in their instantiations of fCOI analyses; while choices were principled given their context, there is currently no empirical data on how such analytic choices differently influence outcomes. For example, researchers make different analytic choices as to how to avoid redundancies in the analysis or errors of non-independence (Vul et al., 2009; Vul and Kanwisher, 2010). The problem of non-independence in analyses, or using the same data to define a region of interest and test for that same effect, was brought to the forefront of cognitive neuroscience by the fMRI community over a decade ago (Vul et al., 2009). In order to avoid such analysis errors, one must define regions of interest on data that is independent from that used in the statistical tests.^1^ Both of the fNIRS studies mentioned above used independent subsets of the data from a single task to independently define COIs and test their hypotheses (Hyde et al., 2018; Powell et al., 2018). Hyde and colleagues (2018) used individual subject run-level data, applying a hold-out procedure to leave a run of data out and define the COI to be used for the analysis of the left-out run from the average of all the other runs of that particular participant (see Fig. 1). This was done iteratively for every run, so that the COI for each run was made on independent data from the same participant. Powell and colleagues (2018) used a different method, dividing trials from each of their two conditions into halves (condition 1-half 1, condition 1-half 2, condition 2-half 1, condition 2-half 2) and made 4 contrasts from the unique combination of the subsets (c1h1 > c2h1; c1h2 > c2h2; c1h1 > c2ch2; c1h2 > c2h1), iteratively holding one contrast out and using the other 3 contrasts to define the COI for the held-out contrast (see Fig. 1d). To date, it is unclear how these and other analytic choices on sub-setting data to avoid non-independent errors influence results, as there have been no direct comparisons for these different approaches to fCOI defining and testing on the same dataset(s).

The researchers who conducted the studies mentioned above seemed to have arrived at similar analytic choices for other aspects of the fCOI analysis. For example, both of the reported studies above chose to anatomically constrain the fCOI search to a particular region of the head probe based on best estimates of scalp-brain correspondence. Although this was done based on a priori ideas about where the effect should be found, it assumes that scalp channel-brain correspondences are fairly accurate. It is an open and empirical question how and whether results would differ if the fCOI search was not constrained to particular regions of the head probe. That is, are fCOI results more robust when applying hypothesized anatomical constraints (when they exist) or not?

As another example, researchers in both of the example studies mentioned chose to have the fCOI defined by the single channel showing the strongest predicted responsive. In these cases, researchers were expecting a fairly focal response and placed their probes accordingly. However, an open question is if results would differ, for better or worse, if the fCOI was defined as the average of multiple channels rather than a single channel?

To better understand the implications of these different methods and analytic choices on infant NIRS data analysis, we evaluate their outcomes for two infant fNIRS datasets. For both datasets, we first compared results from a traditional fixed-array analysis approach to various types of fCOI analysis approaches. A traditional fixed-array approach was used as a baseline of comparison from which to evaluate various fCOI methods and choices, given it underlies many foundational findings in the infant fNIRS literature and shares similar fixed “channel-space” assumptions with many other commonly used methods. We then evaluated the effects of different analytic choices on the results for each fCOI method individually. Finally, to further validate our results, we compared these same analytic approaches and choices on a synthetic infant fNIRS dataset, where the ground truth was known. The overarching purpose of comparing and contrasting these different analyses is to better understand their effects on results and to provide a novel empirical basis for analytical decision making in infant fNIRS data analysis moving forward.

## 2. Study 1

### 2.1. Materials and methods

In a first study, we compared and contrasted methods of interest and analytic choices on an infant fNIRS experiment designed to identify face-sensitive regions in the posterior temporal lobe. The dataset was comparable in nature to a previous methodological study of infant fNIRS fCOI analyses (Powell et al., 2018) and similar to dynamic face-specific region localizers used in fMRI research with adults (Pitcher et al., 2011).

#### 2.1.1. Participants

A total of 20 participants made up the final dataset (*M*_*age*_ = 8.65 months, *SD*_*age*_ = 1.10 months, *Range*_*age*_ = 6.31–10.59 months, 7 females). An additional 18 infants participated but did not contribute useable data (became fussy or inattentive before the end of the first block, *n* = 8; unable to obtain good signal due to hair or other probe fit issues before experiment started, *n* = 2; head probe shifted or was pulled by the infant during the experiment, *n* = 6; distracted by the experimenter, *n* = 1; technical failure to record NIRS data during session, *n* = 1).

The study was conducted with approval of the University of Illinois Office for the Protection of Human Subjects. Written informed consent was obtained from a parent or guardian of all children before data collection began. Families were given a small token of appreciation (book or t-shirt) or a travel reimbursement of 10 dollars for their participation.

#### 2.1.2. Stimuli & design

**Fig. 2.**
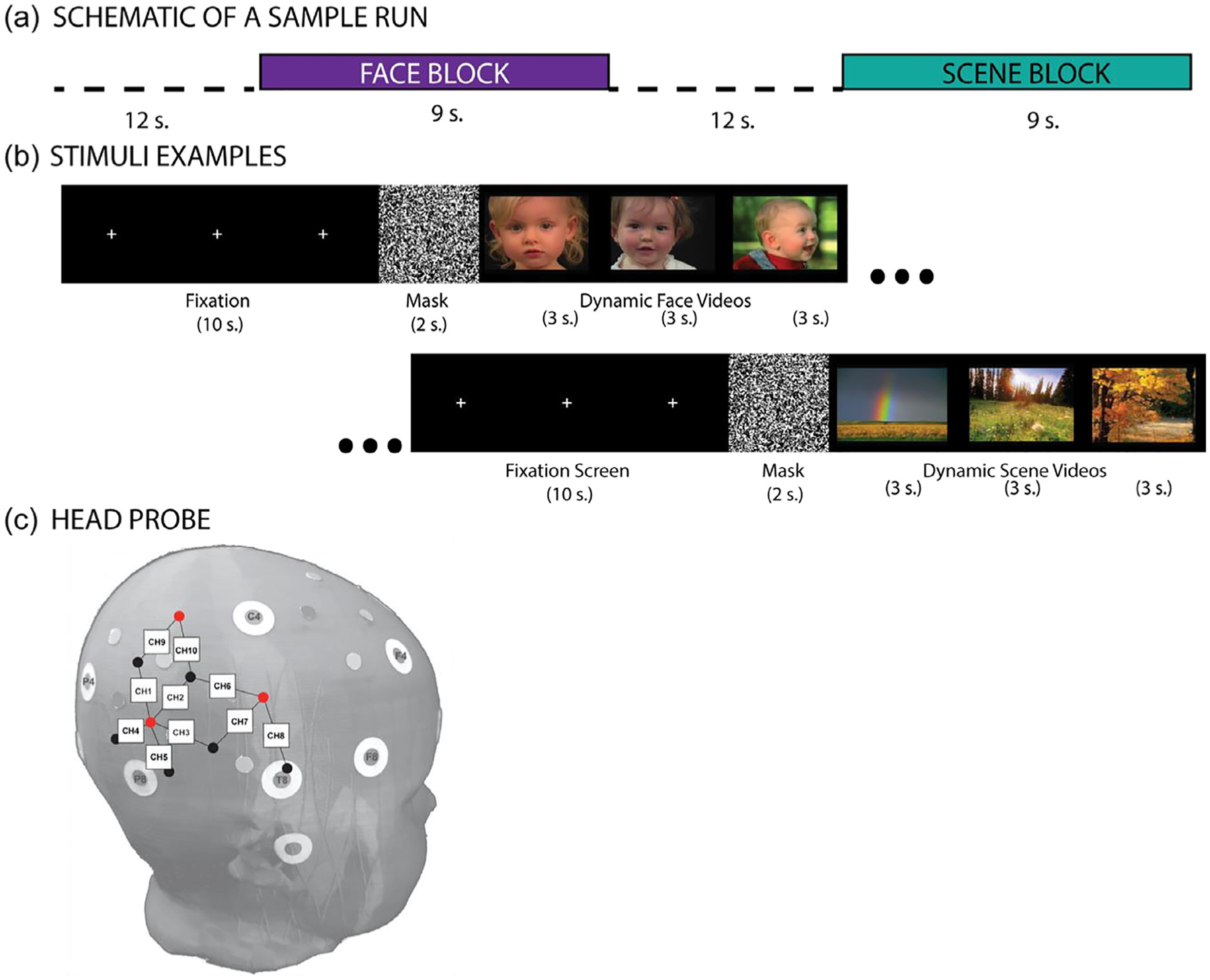
Depiction of stimuli, presentation sequence, and fNIRS probe placement for Study 1. (a) Schematic depiction of timing for a single run. (b) Examples of stimuli used. After fixation and mask, three 3 s dynamic videos of faces or scenes were presented. Picture represent still images extracted from videos. (c) Depiction of temporal head probe positioning relative to common scalp landmarks. White circles represent 10–10 scalp landmarks. Red circles represent light sources and black circles represent light detectors. Black lines connecting light sources and detectors represent data channels. Square boxes with numbers are used to label each data channel 1–10.

Stimuli were designed to elicit activity in regions of the lateral temporal cortex selective to dynamic faces (Powell et al., 2018; also see Pitcher et al., 2011). They were comprised of dynamic movies featuring either faces or landscape scenes (see Fig. 2). Dynamic face videos involved a child moving around naturally, with the video focused on the face (shared and adapted with permission from Pitcher et al., 2011). Dynamic scene videos involved fly-through vistas of natural landscapes such as mountains or forests. All video clips were accompanied by instrumental classical music intended to further sustain engagement of infants (Powell et al., 2018). Each run began with a 10-second black screen with a white fixation cross in the middle, followed by 2-second mask screen with an alerting sound played on onset, a blocked sequence of three, 3-second video clips of one type (faces or scenes), another 10-second fixation and 2-second mask, and another blocked sequence of three, 3-second video clips of the other type (faces or scenes, see Fig. 2).

There were 12 unique videos of each type used throughout the experiment. Individual video clips were presented in a unique order for every subject. The order of video type per run (i.e., whether face or scene videos were presented first) was randomized for each subject but fixed throughout the experiment. Specific face and scene video clips within the run were chosen at random without replacement for two runs, after which all videos were replaced and randomization without replacement happened again. This means that all videos in the first two runs were unique and after two runs some individual video clips may have been repeated. Infants were shown up to 8 runs, each run containing a face block and a scene block.

#### 2.1.3. Procedure

The procedure for obtaining data was similar to that we and others have used in past to collect infant fNIRS studies using audiovisual stimuli (Edwards et al., 2016; Hyde et al., 2018; Powell et al., 2018). After obtaining consent, infants were fitted with flexible, custom-made headgear while sitting in the lap of a parent or guardian. Once the headgear was properly fitted and adjusted for signal quality (details below), overhead lights in testing room were shut off, and video clips were presented on a computer monitor approximately 90 cm away from the infant. Through a closed-circuit camera we monitored looking behavior in real time from an adjoining room. After each run was completed, the display paused until the researcher initiated the next one by pressing a key on a keyboard located in the monitor room. Stimuli were presented until the infant became inattentive, fussy, or after seeing the maximum number of runs (defined above).

FNIRS recordings were made using a TechEn CW6 (continuous wave) NIRS system with eight light (four 690 nm and four 830 nm wavelength) sources and four light detectors to measure the cortical hemodynamic response at 50 Hz from the scalp. Sources and detectors were arranged with a fixed spacing of 2.5 cm to create a custom 10-channel probe covering scalp regions corresponding to lateral temporal and inferior parietal regions of the right hemisphere (Fig. 2c). The probe was attached to a custom-made spandex cap and sources and detectors were held in place by tight-fitting rubber grommets. Additionally, sports headbands and Velcro were used as needed to steady the head probe against movement and ensure that optodes remained firmly against the scalp during the procedure. Ten-foot optical fibers carried light to and from the system and the headgear.

Probes were placed relative to common scalp landmarks that correspond to known 10–10 system points (Fig. 2c). More specifically, the anterior-most detector of the temporal portion of the probe was always anchored directly above the preauricular point, as close to the T8 10–10 point (T4 in 10–20 points) as possible with accommodation for the ear; the posterior-most source was then positioned toward the P4 landmark (Fig. 2c). Based on comparison to available fNIRS–MRI scalp atlases (Lloyd-Fox et al., 2014; Matsui et al., 2014), our probe covered scalp regions associated with the right superior temporal sulcus (rSTS), including posterior and middle superior temporal regions. Signal strength and quality at each channel was adjusted at the beginning of the experimental session and monitored as needed so that an attenuation value of the recording was between 60 and 140 db for at least 75% of the channels.

#### 2.1.4. Data analysis

##### 2.1.4.1. Data pre-processing.

Data pre-processing was conducted using the freely available Homer2 package (version 2.8; Huppert et al., 2009) in MATLAB (R2019a). Our pre-processing steps were closely matched to those used in two recently published infant fNIRS experiments with the same fNIRS system, a similar temporal probe, a comparable participant age range, and similar video-based stimuli (Hyde et al., 2018). First, we began by isolating each run of the experiment from the continuous data to process as a separate file so that normalization of a given run was not skewed by more or less noisy portions of the data across the time course of the experiment. Raw intensity data from each channel in each run was then subjected to an automatic pruning algorithm to identify and reject any channel with too high of a signal (mean intensity > 1 × 10^7)^, too low of a signal (mean intensity < = 0), insufficient signal to noise ratio (mean intensity/standard deviation of intensity < 2), or source-detector separation of less than 2 or greater than 3 cm from further analysis. Intensity data from each run was then normalized and converted to optical density units. We then applied a principal components analysis-based (PCA) filter (as implemented in Homer2, Huppert et al., 2009) to objectively identify and filter out changes in the signal pervasive across the probe, such as movement-induced signal changes, with the constraint to remove no more than 80% of the total variance of a given subject’s data on that run (Cooper et al., 2012; Zhang et al., 2005). A 0.5 Hz low pass filter was then applied to remove higher frequency noise. Then, another automatic algorithm was applied to detect and reject remaining data containing further evidence of movement artifact. Portions of the data with a change in the signal greater than 0.3 optical density units over a given sample (0.2 s) were masked from 2 s before to 2 s after the data sample(s) containing the artifact (Cooper et al., 2012; Huppert et al., 2009; Scholkmann et al., 2010; Strangman et al., 2003). Blocks were defined as the response from - 2 s before to 16 s after onset of the first video clip of a given type within a run (and encompassing all three videos of that type). Blocks containing artifact masks were rejected from further analysis. Incomplete runs, where all three face and all three scene videos were not presented, due to the experimenter ending prematurely because of fussiness or inattentiveness were also dropped from the analysis. Finally, data were converted from optical density units to oxygenated, deoxygenated, and total hemoglobin concentration changes using a modified Beer-Lambert Law with the default 6.0 PPF in Homer2 (Obrig et al., 2000; Strangman et al., 2002). In addition to the automatic rejection of blocks due to artifact detection and/or the rejection of incomplete runs, participants without at least 2 full runs containing a block of each type of video stimuli were not analyzed, as they would not allow a full comparison of methods.

After data pre-processing, including automatic artifact detection and data/run exclusion, a total of 20 participants made up the final dataset. Only complete runs with available data for both face and scene blocks were retained for further analysis. The number of runs for each subject ranged from 2 to 8 with an average of 4.5 runs per subject (i.e., a run containing block of each condition).

#### 2.1.5. Inferential data analyses

Here we focused our head probe and associated analysis on regions of the brain potentially selective for faces by contrasting the response to dynamic face video clips with the response to dynamic scene video clips (Powell et al., 2018; also see Deen et al., 2017; Pitcher et al., 2011). Accordingly, our statistical tests were one-sided t-tests, where we anticipated the response to faces to be greater than the response to scenes (difference in HbO response to face > scenes between 4 and 16 s after video onset), or face-selectivity. Given the primary purpose of data from this task was to localize dynamic face-selective regions of the posterior temporal lobe for analysis of another task and others have already established the existence of face-selectivity in temporal cortex of young infants (Deen et al., 2017; Powell et al., 2018), we did not develop our head probe to also simultaneously cover place-selective regions. For each *t*-test computed, p-values (see alpha levels below) were used to decide whether a significant effect was observed or not by a given method. We also computed two measures of sensitivity, an effect size via Cohen’s *d* (see Cohen, 1988) and a Bayes Factor representing the strength of the evidence in favor of the alternative hypothesis over the null hypothesis (see Jeffreys, 1998; Morey et al., 2016), to directly compare results across different methods and analytic choices. All analyses were carried out in MATLAB R2019a (The MathWorks Inc.), except for Bayes Factor computation, which was carried out in JAMOVI (The JAMOVI Project, 2021 version 1.6.23).

##### 2.1.5.2. Analysis approaches tested.

In the fixed-array analysis, we analyzed the average response to face blocks relative to average response to scene blocks in all channels separately and report whether any individual channel results reach significance for face-selectivity both at an uncorrected alpha level (*p* <.05) and at a Bonferroni-corrected alpha level of *p* <.005 (0.05/10 channels).

We also conducted four versions of the functionally-defined channel of interest (fCOI) approach to compare and contrast different analytic choices to one another and to the results from the fixed-array analysis. In all these approaches, we were careful to use independent data to define and test for face selectivity by using hold-out procedures, avoiding errors of non-independence in our analyses (Vul et al., 2009). In one version of the fCOI analysis, we defined the COI between-participants using a leave-one-subjectout procedure (Esterman et al., 2010). More specifically, data from one subject was held out and we used data from the average of all other subjects to identify the channel with the greatest face-selectivity (i.e., the most positive value in the difference of the HbO response to faces minus response to scenes). The channel identified was then used as the COI definition from which to extract an average response value for each condition from the held-out subject for further analysis (see Fig. 1a). This hold-out procedure was conducted iteratively for all 20 subjects to determine the channel from which data would be analyzed for each subject. Average response values for each condition for each participant derived by this process were then used for further inferential testing and comparison.

In a second version of the fCOI analysis, we defined the COI within participants using a leave-one-run-out procedure (Hyde et al., 2018). In this case, we held out data from one run of a given participant and used the average of the other runs of that same participant to identify the COI for the held-out run (see Fig. 1b). This was done iteratively for every run for every participant, meaning we determined a COI for every run for every participant using data independent from that run from the same subject. The channel identified was then used as the COI definition from which to extract a response value for each condition from each held-out run. Extracted data from each held-out run were then averaged for each condition for each participant for further inferential testing and comparison.

In a third version of the fCOI analysis, we again defined the COI within participants but this time using a split-half method. With this approach, we split the data for each subject into odd and even runs (i.e., face-odd runs, face-even runs, place-odd runs, place-even runs) and then held out even runs and used the average of the odd runs to identify the COI for the held-out even runs (face-odd runs > place-odd runs)(see Fig. 1c). We then did the reverse, holding out odd runs and using the average of the even runs to define the COI for the held-out odd runs (face-even runs > place-even runs). Average response values derived by this process for each half of the data were then averaged for each condition for each participant for further inferential testing and comparison.

In yet a fourth version of the fCOI analysis, we matched the iterative contrasts method of Powell and colleagues (Powell et al., 2018). With this approach, we divided blocks from each of the two conditions into halves (face-half 1, face-half 2, place-half 1, place-half 2) and made 4 unique contrasts (f1 > p1; f2 > p2; f2 > p1; f1 > p2), iteratively holding one contrast out (e.g., f1 vs. p1) and using the remaining data (e.g., f2 > p2) to identify the COI for the held-out contrast (see Fig. 1d). The identified COI was then used to extract data from the conditions in the held-out contrast. The (four) extracted values were then averaged for each condition for each participant for further inferential testing and comparison.

##### 2.1.5.3. Number of channels included in the fCOI.

We were also interested in knowing whether including multiple channels in the fCOI definition would change the results. To that end, we re-ran all these analyses averaging either the top 2 or top 3 most responsive channels (face > place) and compared the results to those obtained using 1 channel with each of the fCOI variations.

##### 2.1.5.4. Anatomical restriction of channels included in the fCOI.

A final dimension of analytic interest was whether anatomical restriction would change the results. As is common in neuroimaging studies, researchers often restrict their analysis to channels of interest based on the scalp localizations thought to be most sensitive to brain regions of interest based on known or estimated scalp-brain correspondences (Gervain et al., 2008; Ichikawa et al., 2010). We developed our head probe with the intention that light source A would be centered on the portion of posterior STS thought to be selectively respond to dynamic faces based on previous work (see Fig. 1b). In contrast, other parts of the probe were aligned with particular scalp landmarks to increase reliability of placements and source estimates (see procedure details above). To investigate the effects of anatomical restriction, we re-ran our analysis, restricting COI definitions to only the 5 detectors linked to light source A and compared the results to those obtained when all channels were eligible in the fCOI definition.

##### 2.1.5.5. Comparison of methods and different analytic choices.

We compared statistical outcomes between methods using inferential statistics, effect sizes, and a metric of evidentiary strength. More specifically, as a first pass we contrasted methods that produced statistically significant effects with those that did not. To more directly compare sensitivity between methods, we considered the relative magnitude of the effects using Cohen’s *d* and evidentiary strength using Bayes Factors.

We also assessed distributional consistency in COI definitions (within- and/or between-subjects) for each analysis version, although for reasons discussed below these were not a primary metric of comparison between methods. First, we visualized the distribution of fCOIs selected for each method. Second, we calculated the consistency in fCOI definitions between-subjects. We did this by identifying the most frequent channel(s) selected as the fCOI for a given participant (out of all fCOIs chosen for that participant), and then identified the channel or channels most frequently selected as the fCOI between the participants from the pool of each participant’s most frequently selected channel or channels. From this, we calculated the proportion of instances the top channels were selected between participants (frequency of top channels / (number of subjects × number of channels in fCOI definition). Third, we calculated the consistency of fCOI definitions within subjects for the within-subjects analysis approaches. Consistency calculations necessarily varied between methods due to differences in the number of fCOI definitions produced by each method. For the split-half analysis method, consistency was defined as the proportion of identical channels of interest between the two halves for each participant. For the iterative contrasts analysis method, consistency was defined as the proportion of identical channels of interest across the four iterations within each participant (4 x the number of channels in that particular fCOI). For leave-one-run-out analysis method, we first found the most frequently identified channel(s) of interest for a given participant and then calculated the number of runs where the most frequent channel(s) was/were selected out of the total number of runs for that participant (i.e., total runs for a given subject × the number of channels used in that fCOI definition).

### 2.2. Results and discussion

#### 2.2.1. Fixed-array analysis

**Fig. 3.**
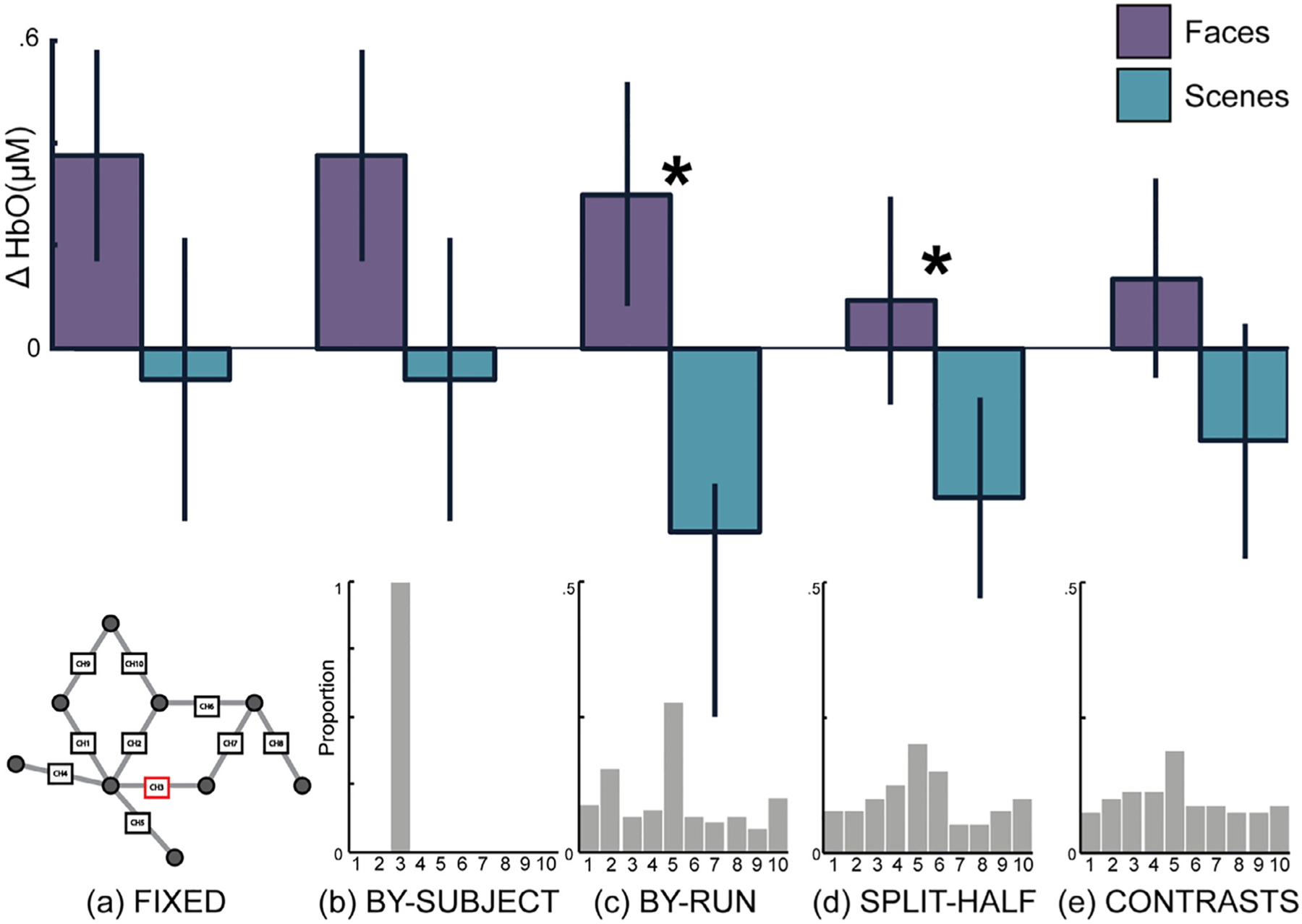
Extracted HbO responses to faces and scenes for each method (top row). Method labels (a–e) can be found at the bottom of the figure, along with proportional distributions of channels chosen as the single-channel fCOI by each methodology. Error bars represent −/+ 1 standard error of the mean (SEM). Note: Only data from Channel 3 is displayed for the fixed-array method(a).

**Fig. 4.**
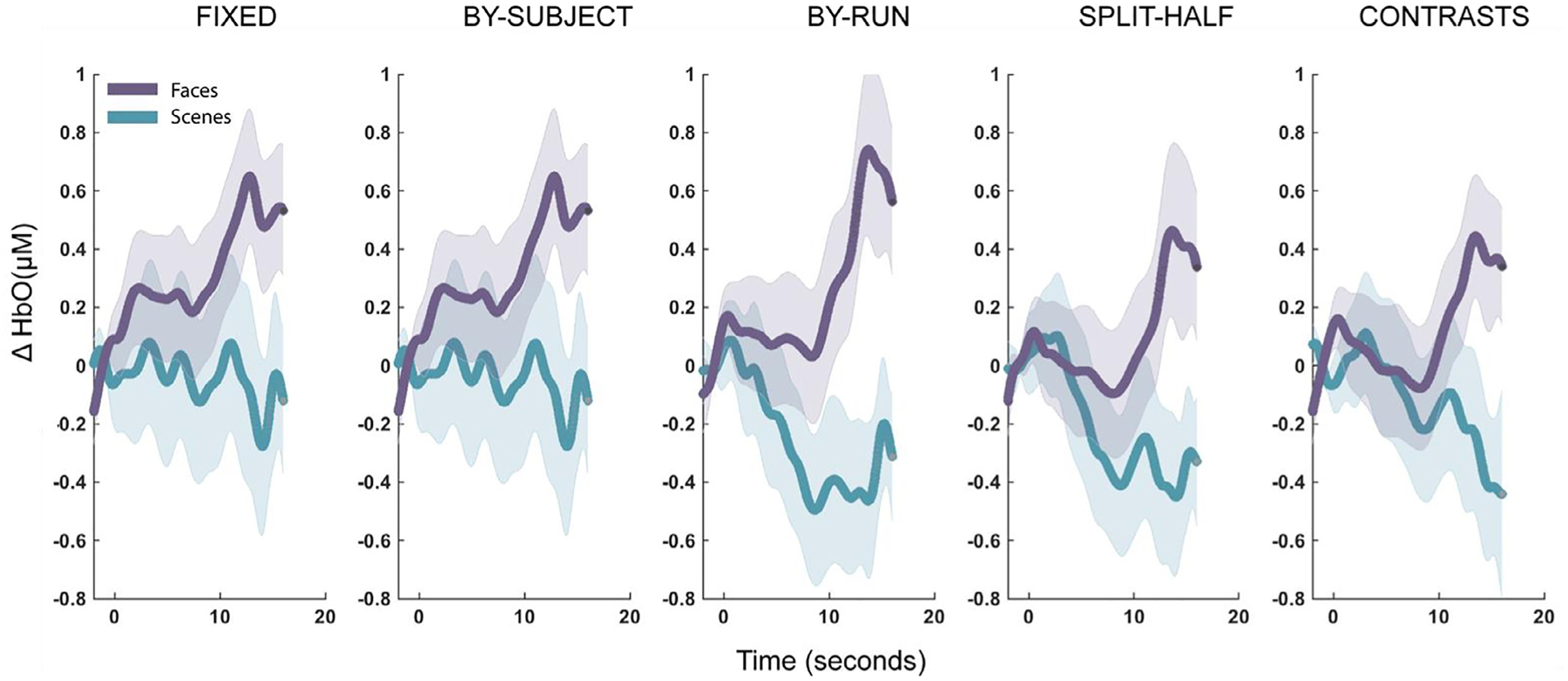
Study 1 HbO time course for each method of analysis with single channel definitions. Dark lines represent group means and shading around lines represents −/+ 1 standard error of the mean (SEM). Data from channel 3 is displayed for the fixed-array method. The a priori window of analysis for all methods was 4–16 s.

A fixed-array analysis contrasting face processing with scene processing at each channel separately did not yield any statistically significant results, even before correction for multiple comparisons (see Table 1). The strongest effect was seen in Ch. 3 (Cohen’s *d* = 0.37), followed by Ch. 7 (*d* = 0.29), and then Ch. 5 (*d* = 0.25), although all corresponding Bayes Factors (BF_10_ = 0.68–1.43) suggested relatively weak evidence for a face effect (see Table 1, Figs. 3 and 4) (Jeffreys, 1998).

#### 2.2.2. Functionally-defined channel(s) of interest analyses

Defining a channel of interest by contrasting the functional response to face processing with scene processing yielded statistically significant results using the within-subjects leave-one-run-out and the split-half hold out procedures, but not using within-subjects iterative contrasts procedure or the between-subjects leave-one-subject out procedure (see Table 2, Figs. 3 and 4). The largest effect was seen with the leave-one-run-out procedure (*d* = 0.51), followed by the split-half procedure (*d* = 0.39). However, the strength of the evidence was about twice as large for the leave-one-run-out procedure (BF_10_ = 3.76) compared to the other fCOI procedures (BF_10_ = 0.65–1.58) and fixed-array approach (BF_10_ = 1.43).

##### 2.2.2.1. Number of channels included in the fCOI.

Increasing the number of channels of interest selected from one to two or three did not substantially change the results (see Table 2). The leave-one-run-out method and the split-half method, the two analytical approaches where face-selectivity effects were observed to be significant with single channel definitions, remained significant with the average of multiple channels (two or three). The related effect sizes increased slightly in strength with more channels for the split-half method (1 channel *d* = 0.37; 2 or 3 channel *d* = 0.44), and the effect sizes decreased slightly in strength with more channels for leave-one-run-out method (1 channel *d* = 0.51; 2 channel *d* = 0.44; 3 channel *d* = 0.39). Neither the between-subjects method nor the iterative contrasts method yielded face-selective significant effects when including multiple channels in the fCOI definition, just as they had not with a single channel definition. Similarly, effect sizes and Bayes Factors were either maintained or slightly reduced for the between-subjects method and the iterative contrasts method when moving from single channel to multiple channel fCOI definitions (see Table 2).

##### 2.2.2.2. Anatomical restriction of channels included in the fCOI.

A final dimension of analytic interest was whether anatomical restriction would change the results. Anatomical restriction improved sensitivity of the split-half method (e.g., 1 channel fCOI w/o restriction *d* = 0.39, BF_10_ = 1.58; 1 channel fCOI w/ restriction *d* = 0.58, BF_10_ = 6.64, see Tables 2 and 3 for full results). However, anatomical restriction either resulted in unchanged or reduced effect sizes for the all the other methodologies (see Tables 2 and 3).

#### 2.2.3. Consistency in channel selection

##### 2.2.3.1. Between-subject consistency.

Defining the fCOI for each subject using data from the other subjects (i.e., leave-one-subjectout) resulted in a high level of consistency in the channel selected as the COI between subjects. With a single channel of interest, Ch. 3 was selected as the COI for all subjects (100%, see Fig. 3). When the COI definition was expanded to two channels, Ch. 3 and Ch. 5 were selected 95% of the time. Ch. 3, Ch. 5, and Ch. 1 were the most common when the COI definition included three channels, being selected 86.67% of the time.

As one might expect, there was less consistency between-subjects when the COI(s) were defined within subjects, as this method allows flexibility based on functional data from each individual. Nevertheless, the most frequent channels selected as COIs were largely consistent with the between-subjects method. For all within-subject selection methods, Ch. 5 was shown to be the most frequently selected COI with single channel definitions (27.78% leave-one-run-out; 20% by-half; 18.75% iterative contrasts, see Fig. 3 for full distributions). For two channel definitions, Ch. 3 and Ch. 5 emerged as the most frequent channels selected (out of all channels selected for all participants: 30.00% of runs; 36.25% of halves; 33.75% of iterative contrasts). For three channel definitions, Ch. 3, Ch. 5 and Ch. 8 were selected most frequently for the iterative contrasts (41.25% of all contrasts) and split-half method (40.83% of all halves) methods and Ch. 3, Ch. 4 and Ch. 5 were selected most frequently for the leave-one-run-out method (41.85% of all runs)..

##### 2.2.3.2. Within-subject consistency.

For within-subjects leave-one-run-out procedure, the same channel was identified for the same subject 70.95% of the time. Within-subject reliability increased to 77.17% when two channels of interest were used and increased further to 80.34% when three channels of interest were used as the definition (varied between two and eight runs per subject). For the within-subjects split-half procedure, the same channel was identified for the same subject 15% of the time. Within-subject reliability increased to 30% when two channels of interest were used and further increased to 38.33% when three channels of interest were used as the definition. For the within-subjects iterative contrasts procedure, the same channel was identified for the same subject 52.5% of the time. Within-subject reliability increased to 64.38% when two channels of interest were used and further increased to 70.42% when three channels of interest were used as the definition.

### 2.3. Discussion

Conceptually replicating a previous study (Powell et al., 2018), we found evidence that fCOI approaches provide greater sensitivity for the detection of face-selective regions than a traditional fixed-array analysis. More specifically, only fCOI approaches showed statistically significant effects and fCOI methods resulted in substantially larger effect sizes compared to the fixed-array approach. As evidenced by effect sizes and evidentiary strength, we found that within-subjects fCOI approaches overall were more sensitive to the effect of interest than a between-subjects fCOI approach. Furthermore, among the three within-subjects fCOI approaches, the leave-one-run-out method yielded the strongest results, followed by the split-half method, and then the iterative contrasts method. Adding multiple sites to the fCOI definition (2 or 3) did not substantially improve sensitivity and did not change whether the results yielded were statistically significant or not. Anatomically restricting to posterior channels also did not universally improve the results. For most of the fCOI methods, applying anatomical restriction resulted in the same or slightly diminished effect sizes (but see split-half method).

A consistent picture emerged across all approaches in which particular data channels were identified as the channels of interest and these channels aligned with those showing the strongest effects in the fixed-array analysis. There was also some degree of within-subject consistency in the channels selected for the fCOI definition, although overall values were lower than one might expect. This suggests to us that the response to face stimuli possibly spanned multiple channels of the probe even within a single participant, leading to within-subject variability in fCOI definitions across different subsets of the data (as also suggested by Powell et al., 2018). However, it is important to note that calculations of consistency may themselves be challenging with the nature and amount of data available from our study (e.g., split-half method of within-subject consistency = 0% if fCOI for two halves are not the same). More broadly, both the distribution of target channels between subjects as well as metrics of sensitivity could have been amplified by the relatively large age range (and correspondingly larger variance in head sizes and probe fit) of this particular sample and a narrower age range may not necessarily yield the same results.

## 3. Study 2

In a second study, we re-analyzed data from a previously published study to further compare and contrast the same methods and analytical choices. More specifically, we combined data from two infant fNIRS experiments (*N* = 50) on temporal-parietal sensitivity to mental states of others, or theory of mind (Hyde et al., 2018). Across two studies, an original study and a pre-registered replication, the authors found evidence of right posterior temporal-parietal sensitivity to mental states in infants using fNIRS. The nature of these data were different than that of Study 1, as functional responses were extracted from brief time windows synched to particular events within a video stimulus rather than averaged over a larger block of time encompassing multiple video clips. In addition to the type of data, the amount of data per subject was also different (see below). Furthermore, unlike Study 1, we had a priori knowledge of the timing and localization of the effect of interest from the previously published work (Hyde et al., 2015, 2018), allowing us to further evaluate the accuracy of different methods and choices relative to the published findings.

### 3.1. Materials and methods

The methods used to acquire and pre-process data for this study have been previously published in Hyde et al. (2018) and are nearly identical to those used in Study 1. Below we highlight those aspects of the procedure, pre-processing, and analysis that differed from Study 1.

#### 3.1.1. Participants

A total of 50 participants made up the available dataset of participants included over the two experiments (*M*_*age*_ = 7.64 months, *SD*_*age*_ = 0.83 months, *Range*_*age*_ = 6.08–9.07 months, 25 females). The study was conducted with approval of the University of Illinois Office for the Protection of Human Subjects. Written informed consent was obtained from a parent or guardian of all children before data collection began.

#### 3.1.2. Stimuli & design

**Fig. 5.**
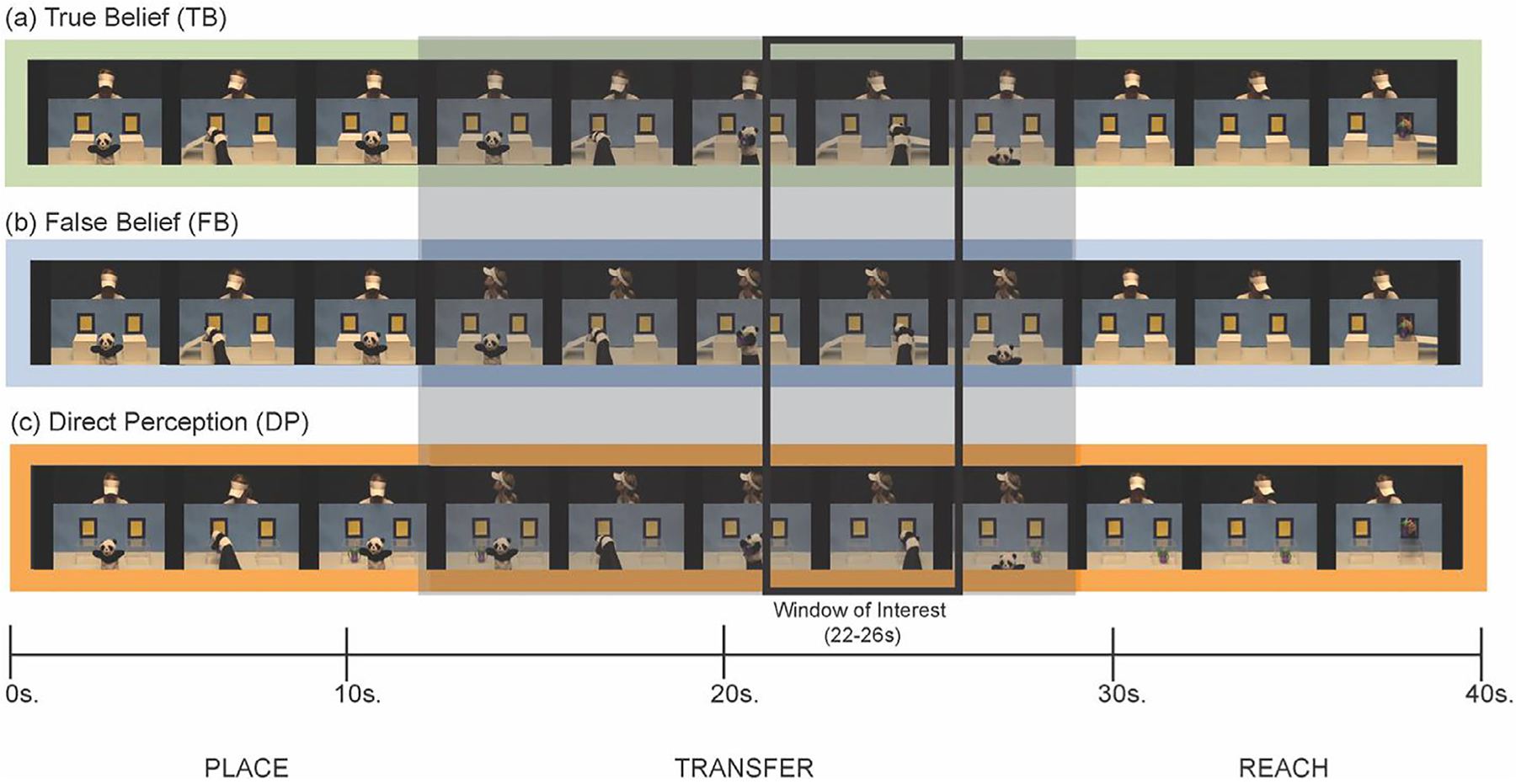
Schematic depiction of video stimuli presented in fNIRS study of infant theory of mind: (a) in the true belief condition, a person watches as a puppet hides an object in one of two boxes and then transfers it to the other box. After the transfer, the person reaches and successfully obtains the object. (b) In the false belief condition, the person watches the puppet hide the object in the first box but turns away before the puppet transfers the object to the other box. After the transfer, the person turns back toward the boxes and reaches successfully for the object. (c) In the direct perception condition, the person makes the same movements as in the FB condition, but the boxes are transparent, giving her access to the object’s location. After the transfer, the person successfully reaches for the object. Below the depictions of the stimuli is an approximate timescale of events as they unfolded through the initial placement phase, the transfer phase, and the final reach phase. The gray box around the transfer phase highlights the time window where the conditions differ conceptually. The smaller black outlined box represents the time window of interest for analyzing the brain response derived from previous work with adults (Hyde et al., 2015). Figure and figure caption reproduced with permission from Hyde et al. (2018).

Stimuli were designed to elicit functional responses in regions of the right temporal-parietal junction (TPJ) thought to selectively respond when adults and older children think about the mental states of other people (Hyde et al., 2018). Stimuli were video clips of a person interacting with a puppet and an object in a goal-directed manner (see Fig. 5). All runs were comprised of an introductory event and followed by three test events presented in a random order. The introductory event was presented to familiarize infants with the particular person, object, and puppet in that particular run. The three test events, or experimental conditions, included a puppet picking up an object, putting it in a box, and then moving the object to another box, as a person stood in the background. All test events ended with the person successfully reaching for the object at the box where it resided. The critical difference between conditions was whether the person knew where the object actually was. In the true belief condition (TB), the person was facing forward and saw the puppet moving the object from one box to another. In the false belief condition (FB), the person had temporarily turned their head away and did not see the object being moved between boxes. In the direction perception control condition (DP), the person had also temporarily turned their head away and did not see the object being moved between boxes, but the boxes were clear (and the location of the hidden object was obvious once they turned back around). A total of four possible runs, each with the three test events (or test trials), were presented involving a new person interacting with a new object each run (see Hyde et al., 2018 for more details).

#### 3.1.3. Procedure

**Fig. 6.**
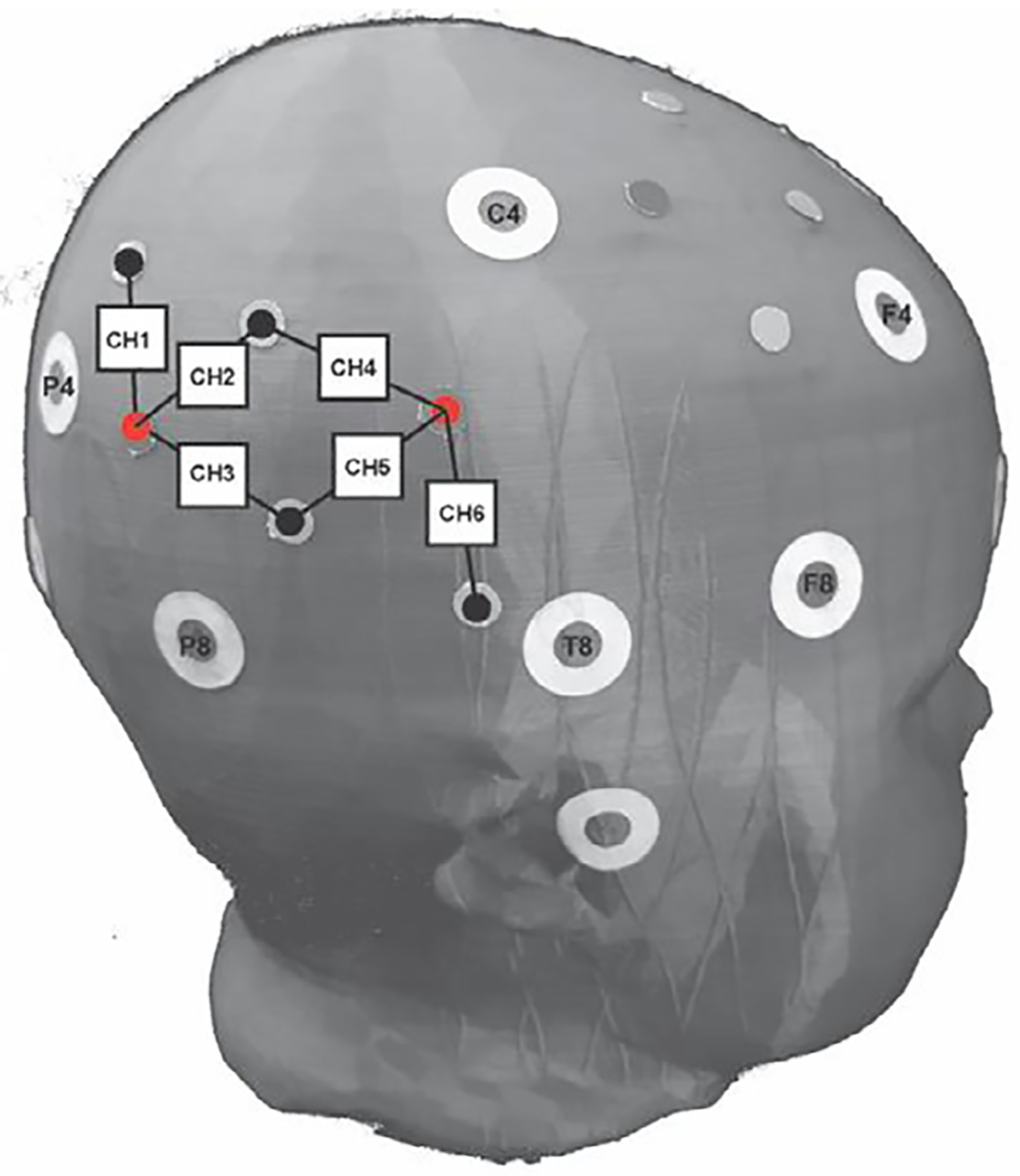
Depiction of temporal head probe positioning relative to common scalp landmarks: White circles represent 10–10 scalp landmarks. Red circles represent light sources and black circles represent light detectors. Black lines connecting light sources and detectors represent data channels. Square boxes with numbers are used to label each data channel 1–6. Figure image and caption partially reproduced with permission from Hyde et al. (2018).

The procedure for obtaining data was very similar to Study 1. Only differences are noted here (see Hyde et al., 2018 for more details). For 20 subjects, sources and detectors were arranged with a fixed spacing of 2.5 cm to create a custom 12-channel probe covering scalp regions corresponding to inferior parietal, lateral temporal, and lateral PFC of the right hemisphere. For another 30 subjects, the probe covered the same 6 temporal-parietal channels on the right hemisphere and 4 channels over the left frontal region. For the purposes of this analysis, we combined data from the two studies and only analyzed the six-overlapping temporal-parietal channels from both experiments (see Fig. 6).

#### 3.1.4. Data analysis

##### 3.1.4.10. Data pre-processing.

Data pre-processing matched exactly that of the original publication of this dataset (Hyde et al., 2018) and was also very similar to the procedure used in Study 1, with the following exceptions. The PCA filter was set to take out no more than 90% of the total variance of a given subject’s data instead of 80% as in Study 1 to match the published processing stream. Trials were defined as 2 s before to 42 s after onset of the test video, to accommodate the full response to the particular stimuli of this study.

#### 3.1.5. Data exclusion

In the published study, participants who completed at least one run behaviorally, which included viewing all three test conditions at least once, without becoming upset and for whom any remaining artifact-free data were retained after preprocessing were included for potential analysis. This yielded a dataset of 50 participants for consideration, with an average of 2.06 trials per condition per participant. When participants with less than two complete runs were excluded from analysis, a requirement for our fCOI analyses below, 34 participants remained with an average of 2.41 trials per condition retained.

#### 3.1.6 Inferential data analyses

We established our time window and functional response pattern predictions directly from the previously published work. Specifically, we focused on the right TPJ response between 22 and 26 s after test video onset as in published studies with this dataset and others (Hyde et al., 2015, 2018). It was also predicted directly from the published work that the response to the false belief condition would be larger than both the direct perception condition and the true belief condition (Hyde et al., 2015, 2018) in the right TPJ region. To reduce the number of statistical contrasts needed to obtain this functional signature between the three conditions (FB > DP and FB > TB and DP = TB) for our purposes here, we contrasted the response to false belief condition with the average of true belief and direct perception conditions, a simplification that was also implemented in the previously published work (see Hyde et al., 2018, Study 2). Accordingly, our statistical tests were one-sided t-tests, where we anticipated the response to false belief condition to be greater than the average response to true belief and direct perception (false belief > ((true belief + direct perception)/2)). For the fixed-array analysis, we report whether any individual channel results reach significance at an uncorrected alpha level (*p* <.05) and at a Bonferroni-corrected alpha level of *p* <.0083 (0.05/6 channels).

To conduct anatomically restricted analyses, we only included the three most posterior channels (Ch.1-Ch.3) associated with the most posterior light source of the study-specific probe (see Fig. 6). This was the a priori anatomical restriction used in the analysis of the previously published paper. Because only three channels were candidates for fCOI definitions under anatomical restriction, it only made sense to consider 1 or 2 channels in analyses with anatomical restriction (not all 3). Other than these study-specific details, the fixed-array and functional fCOI approaches, as well as the analytical choices of interest, were implemented in the same way as in Study 1.

### 3.2. Results and discussion

#### 3.2.1. Fixed-array analysis

**Fig. 7.**
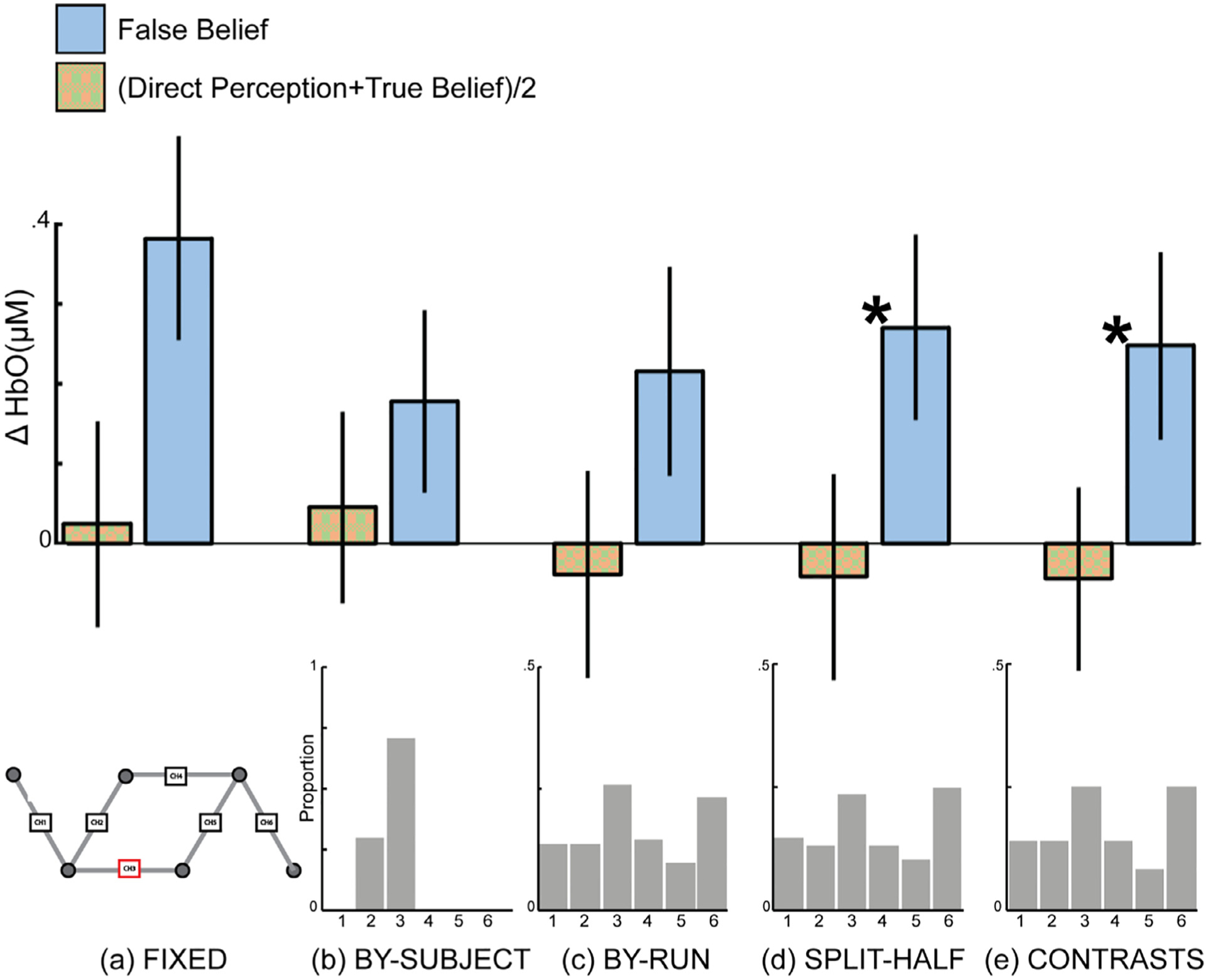
Extracted HbO responses for each method (top row) in Study 2 (for *N* = 34). Method labels (a–e) can be found at the bottom of the figure, along with proportional distributions of channels chosen as the single-channel fCOI by each methodology. Error bars represent −/ + 1 standard error of the mean (SEM). Note: only data from Channel 3 is displayed for the fixed-array method(a).

**Fig. 8.**
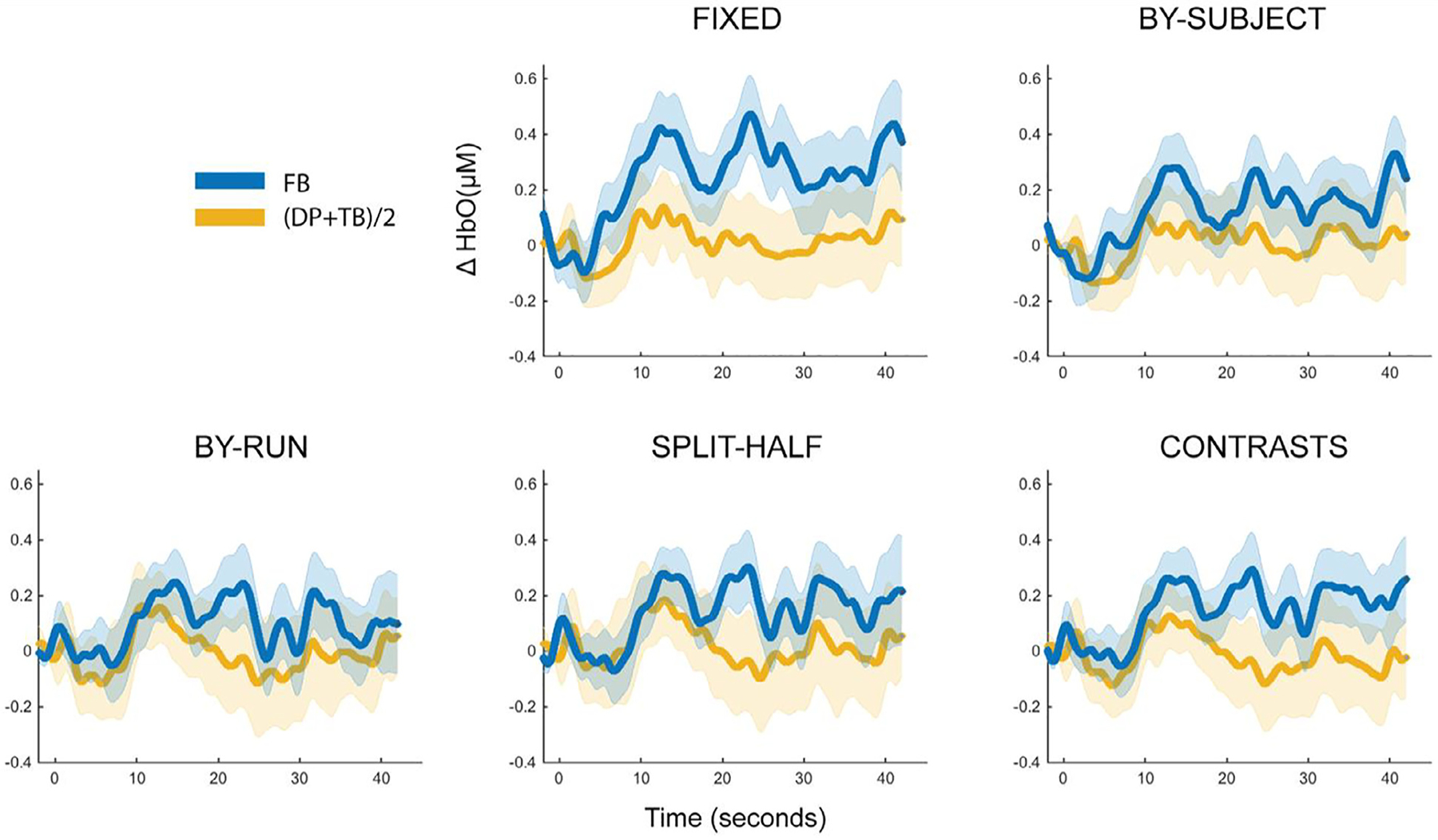
Study 2 HbO time course for each method of analysis with single channel definitions. Dark lines represent group means and shading around lines represents −/ + 1 standard error of the mean (SEM). Only data from Channel 3 is displayed for the fixed-array method. The a priori window of analysis for all methods was 22–26 s.

A fixed-array analysis of the full dataset (*N* = 50) contrasting the response to the false belief condition with the average response from the true belief and direct perception conditions at each channel separately yielded statistically significant effects in Ch. 1, Ch. 2 and Ch. 3, even after correction for multiple comparisons (see Table 4, Figs. 7 and 8). Out of those channels, Ch. 3 showed the largest response (*d* = 0.54) and greatest evidentiary strength (BF_10_ = 140).

Restricting the dataset to only participants with at least two complete runs of data, a constraint required to apply within-subject fCOI approaches, yielded a remaining sample of 34 participants. The same three channels identified in the full dataset as significant (Ch. 1, Ch. 2, & Ch. 3) showed the strongest effects in the restricted sample, although none remained statistically significant after correction for multiple comparisons (see Table 5). The strongest effect was again observed in Ch. 3, with an effect size (*N* = 34, *d* = 0.47) only slightly reduced from the full (*N* = 50, *d* = 0.54) sample. The evidentiary strength for Ch. 3, however, was substantially reduced in the reduced dataset (BF_10_ = 4.62) compared to the full dataset (BF_10_ = 140), most likely due to the difference in the number of participants included.

#### 3.2.2. Functionally-defined channel(s) of interest analysis

Defining a single channel of interest by contrasting the functional response to the false belief condition with the average of the response to the true belief and direct perception conditions yielded statistically significant results using a split-half procedure and iterative contrasts procedure but did not yield a statistically significant effect using between-subjects leave-one-subjectout procedure or within-subjects leave-one-run-out procedure (see Table 6, Figs. 7 and 8). The split-half and iterative contrasts methods produced medium effect sizes (*d* = 0.32–0.34), whereas the between-subjects leave-one-subjectout^2^ and within-subjects leave-one-run-out methods produced smaller effect sizes (*d* = 0.15–0.26). The iterative contrasts method yielded the most robust results, with the largest effect size (*d* = 0.34) and greatest evidentiary strength for differential sensitivity to the false belief condition (BF_10_ = 2.19).

##### 3.2.2.1. Number of channels included in the fCOI.

Increasing number of channels from a single channel of interest to two to three channels did not substantially change the results for the split-half or iterative contrasts methods (see Table 5). Both remained statistically significant with multiple channels, although effect sizes did not substantially increase by increasing the number of channels included in the fCOI definition. In contrast, increasing the number of channels did improve results from the between-subjects leave-one-subject-out and the within-subjects leave-one-run-out methods (see Table 6). Results of the leave-one-subject-out method became significant by using multiple channels, with the two channels showing the largest effect size and strength of evidence for an effect (see Table 6). Results from the leave-one-run-out procedure became significant only with three channels, although effect sizes and strength of the evidence for an effect did not change substantially by including multiple channels in the fCOI. (see Table 6).

##### 3.2.2.2. Anatomical restriction of channels included in the fCOI.

Applying anatomical restriction further improved the leave-one-run-out, split-half, and the iterative contrasts results for both single and multiple channel definitions (see Table 7). Results remained largely the same for the between-subjects method (see Table 7).

#### 3.2.3. Consistency in channel selection

##### 3.2.3.1. Between-subjects consistency.

Defining fCOI for each subject using the data from other subjects resulted in a high level of consistency in the channel(s) selected as the COI between subjects. With a single channel of interest, Ch. 3 was selected as the COI 70.59% of the time (see Fig. 7). When the definition included two channels, Ch. 2 and Ch. 3 were selected 98.53% of the time. Ch. 1, Ch. 2, and Ch. 3 were selected 94.12% of the time when the COI definition increased to three channels.

As expected, between-subjects consistency decreased when the COI(s) were defined within-subjects since this method allows for flexibility based on individual subject functional data. Nevertheless, the most frequent channels selected as COIs were largely consistent with those identified using the between-subjects fCOI method. Using the leave-one-run-out method Ch. 3 was the most frequently selected for single channel definitions (25.61%, see Fig. 7), Ch. 2 and Ch. 3 were selected most frequently for two channel definitions (40.85% of the time), and Ch. 2, Ch. 3, and Ch. 6 were selected most frequently (57.32%) for three channel definitions. For the split-half method, Ch. 6 emerged as the most frequent (25%, followed closely by channel 3 with 23.53%) with single channel definitions (see Fig. 7), Ch. 2 and Ch. 3 were most frequent (39.71%) for two channel definitions, and Ch. 2, Ch. 3, and Ch. 6 were most frequent (57.36%) for three channel definitions. For the iterative contrasts method, Ch. 3 was tied for the most frequent (25%) with Ch. 6 (25%) for single channel definitions (see Fig. 7), Ch. 2 and Ch. 6 were most frequent (40.07%) for two channel definitions, and Ch. 2, Ch. 3, and Ch. 6 were most frequent (56.86%) for three channel definitions.

##### 3.2.3.2. Within-subjects consistency.

For the within-subjects, leave-one-run-out procedure, the most frequent channel was identified an average of 59.80% of the time within a given subject. Within-subject consistency was 68.63% when two channels of interests were used and further increased to 75.41% when three channels of interests were used as the COI definition. For the within-subjects split-half procedure, the same channel was identified for the same subject 17.65% of the time. Consistency increased to 35.29% when two channels of interests were used and further increased to 50.98% when three channels of interests were used as the COI definition. For the within-subjects iterative contrasts procedure, the same channel was identified for the same subject 58.09% of the time. Within-subject consistency increased to 67.65% when two channels of interests were used and further increased to 75.49% when three channels of interests were used as the COI definition.

### 3.3. Discussion

In Study 2, we contrasted the same analytical approaches and decisions used in Study 1 with a different, infant fNIRS dataset to further understand their resulting effects. More specifically, we applied these analyses to a previously published dataset where we had investigated and found evidence that a posterior temporal-parietal region of the infant brain was sensitive to the belief of another person (portrayed in a video) about the location of a hidden object, or theory of mind (see Hyde et al., 2018).

Broadly as in Study 1, we found evidence that fCOI approaches were more sensitive to the effects of interest compared to the fixed-array approach and within-subjects fCOI approaches were more sensitive than the between-subject fCOI approach. Of the within fCOI approaches, the iterative contrasts and split-half method yielded the strongest results with this dataset. Including multiple channels (2 or 3) in the fCOI definition did not weaken the result and including multiple channels appeared to increase effect sizes for the initially less sensitive between-subjects fCOI method and the within-subjects leave-one-subject-out methods. Anatomical restriction improved the within-subjects leave-one-run-out, split-half, and iterative contrasts methods, but had no effect on the between-subjects leave-one-subjectout method.

Although there was clearly a distribution of top channels chosen, the most frequent channels identified as sensitive to the effects of interest seemed to be largely consistent across methods (see Fig. 7). Furthermore, the most frequently identified fCOI channel, Ch. 3, was consistent with the localization of the effect seen in the previously published work with this dataset, further validating the analysis approaches used here (Hyde et al., 2018). A calculation of within-subject consistency, although not directly comparable across approaches, yielded comparable if not higher values than those observed in Study 1. Although, as suggested above, consistency metrics may themselves be challenging to calculate and compare with these sorts of data and these problems may even be exacerbated with the nature and minimal amount of data available in Study 2.

Taken all the evidence together from Study 2, the iterative contrasts method and the split-half method performed well in terms of sensitivity. The within-subjects leave-one-run-out method, at least without anatomical restriction or multiple channels, appeared less sensitive with this particular dataset compared to other fCOI methods.

One major constraint we discovered with this dataset that was not as evident in Study 1 was that the within-subjects fCOI approaches require at least two acceptable runs per subject for analyses, reducing 50 potential subjects to 34. While a comparison of sensitivity using the same number of subjects (i.e., the reduced dataset) provided evidence that within-subject fCOI methods are more sensitive to the effects of interest than a fixed-array analysis or the between-subjects fCOI analysis all else being equal, this finding must be qualified. If all useable subjects were included for the fixed-array analysis or the between-subjects fCOI analysis, methods requiring only a single complete run of data, resulting sensitivity was similar to the within-subject fCOI methods. Thus, our results show that the fixed-array and between-subjects methods can yield comparable sensitivity to within-subjects fCOI analyses if the number of subjects is increased. In our sample, this increase in sample size was around a 50% increase.

## 4. Study 3

While the primary purpose of our investigation is to compare and contrast results from fixed and fCOI methods with real infant fNIRS datasets, including their particular idiosyncrasies, a limitation of doing so is that the ground truth of the signal under investigation is unknown. To further validate these techniques and better understand our results, we compared and contrasted the same methods and analytical choices on a synthetic infant fNIRS dataset where the ground truth was known.

### 4.1. Materials and methods

#### 4.1.1. Data simulation settings and design

Using tools from the NIRS Brain AnalyzIR Toolbox (Santosa et al., 2018) as well as publicly available code for infant-specific fNIRS data simulation (Gemignani and Gervain, 2021a), we created a synthetic dataset of 20 participants. For this simulation, we used the same basic 10 channel head probe as in Study 1 (see Fig. 2c) with two conditions in the design, an active condition and a control condition. Conditions mimicked a slow block design, occurring in alternation and separated by random interval of 45–51 s

The synthetic fNIRS data was created following recommendations in the literature for simulating infant-specific fNIRS data (see Gemignani and Gervain, 2021a). First, spatially and temporally correlated baseline noise was generated using an autoregressive model (order = 30; spatial correlation = 0.33). Next systematic components simulating heart rate (1.5 Hz), respiration (0.3 Hz), Mayer waves (0.1 Hz), were added to the baseline noise with settings to simulate infant-specific physiology (Gemignani and Gervain, 2021a). Then, a canonical hemodynamic response function (HRF) for the active condition (Santosa et al., 2018, 4 s to peak, 32 s duration with 16 s undershoot) was inserted in 2 channels for each subject. The scale of the HRF was set on the large end of the range of suggested infant HRFs (beta = 0.35 for active condition, Gemignani and Gervain, 2021a) and a negative HRF was set for the control condition (beta = −0.05) in an attempt to produce a prominent HRF difference between conditions detectable by all analysis methods. However, the particular two channels chosen to receive the HRF varied randomly for each participant between Channels 1–3 in the head probe to simulate variability in active channels between participants, a data feature critical to testing sensitivity and validity of our fCOI identification methods. Finally, simulated motion artifacts were inserted at the frequency (2 Hz spikes; 1.5 Hz shifts) approximating those seen in real infant fNIRS data (Gemignani and Gervain, 2021a).

Our settings were chosen to reasonably approximate a robust infant fNIRS dataset with a localized and prominent HRF response. It is important to note that parametric variation of other settings (beyond channel location) in simulated data, while possibly important and informative for future work, is beyond the scope of this investigation. Critically for our purposes, the same synthetic dataset was fed into all analysis methods reported below and, as such, differences in outcomes between analysis approaches and choices are not likely due to the particular noise, artifact, and HRF settings chosen here.

#### 4.1.2. Data analysis

##### 4.1.2.1. Data pre-processing.

Data pre-processing settings mirrored those of Study 1 with the following changes due to differences in the nature of the synthetic data and design. A 0.7 Hz low pass filter (rather than 0.5) was applied to remove higher frequency noise based on suggestions from Gemignani and Gervain (2021b). Data blocks were defined as - 2 s before to 20 s after onset of condition marker to capture to the synthetic HRF. After preprocessing involving the detection and rejection of trials containing artifacts, an average of 6.95 active condition trials and 7.4 control condition trials remained for analysis.

##### 4.1.2.2. Inferential data analyses.

The mean amplitude response was extracted from 2 to 14 s after condition marker onset to capture the HRF for further analysis. Our inferential statistical tests were one-sided t-tests, where we anticipated the response to the active condition to be greater than the response to control condition (active > control). Corrected and uncorrected alpha levels were the same as those used in Study 1 (*p* <.005, *p* <.05). In addition to distributional, between-, and within- participant consistency metrics in fCOI definitions used in Study 1 and Study 2, we also evaluated the correspondence between the observed fCOI definitions and ground truth channels where the HRF was inserted.

### 4.2. Results and discussion

#### 4.2.1. Fixed-array analysis

**Fig. 9.**
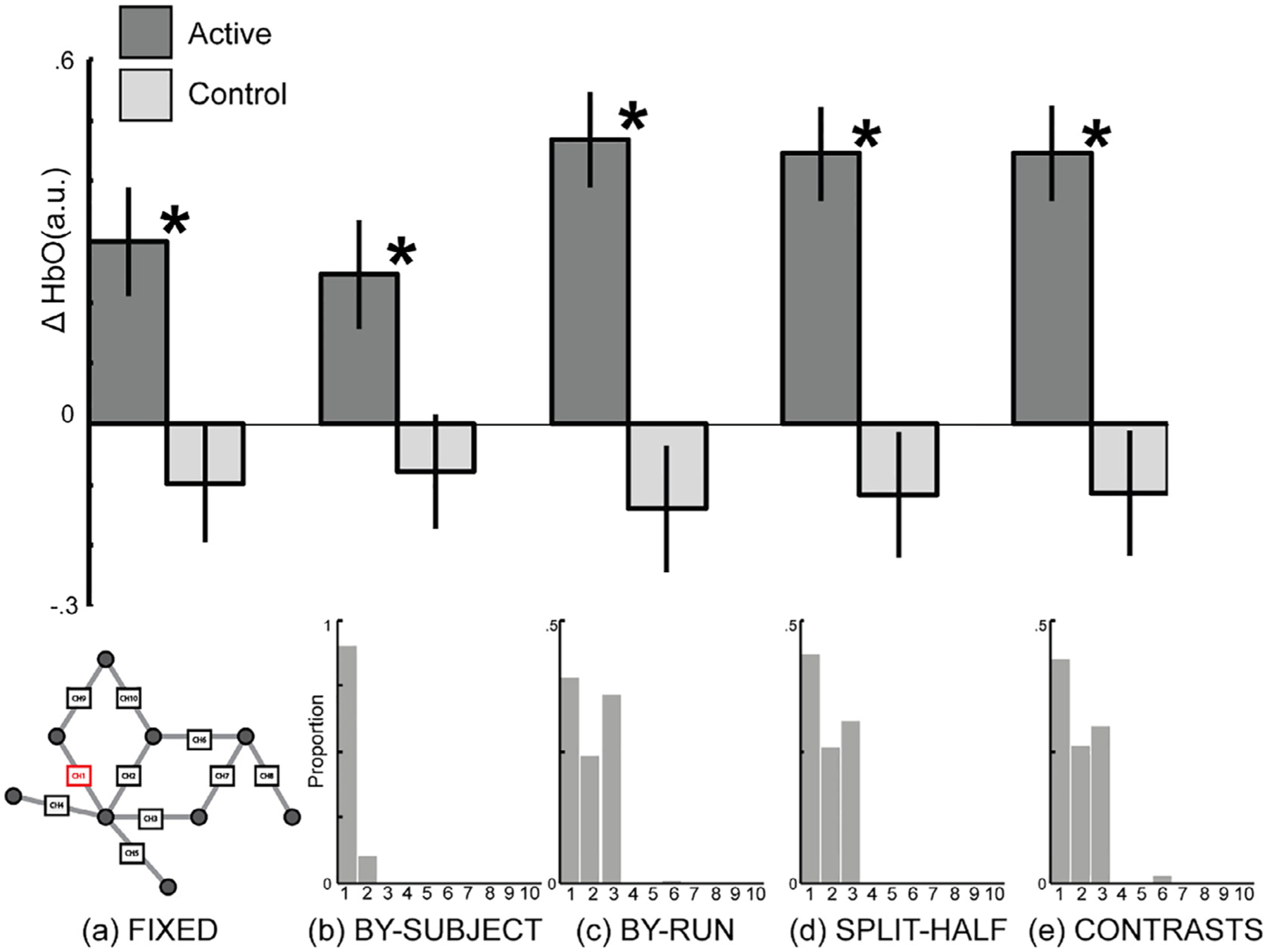
Extracted HbO responses to active and control conditions in simulated infant fNIRS data for each method (top row) in Study 3. Method labels (a–e) can be found at the bottom of the figure, along with proportional distributions of channels chosen as the single-channel fCOI by each methodology. Error bars represent −/+ 1 standard error of the mean (SEM). Note: Only data from Ch. 1 is displayed for the fixed-array method(a).

**Fig. 10.**
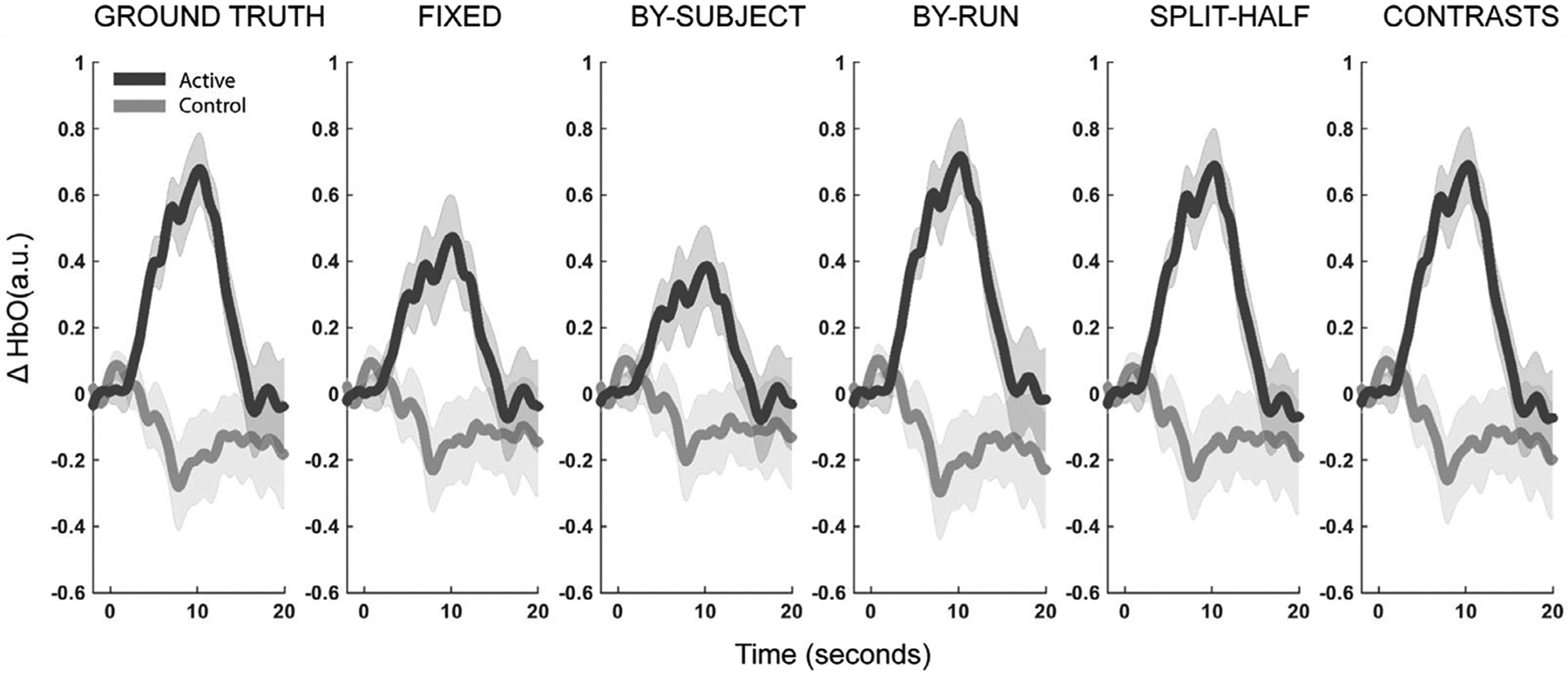
Study 3 HbO time course for ground truth data as well other methods of analysis using single channel definitions. Dark lines represent group means and shading around lines represents −/+ 1 standard error of the mean (SEM). Data from channel 1 is displayed for the fixed-array method. The a priori window of analysis for all methods was 2–14 s.

A fixed-array analysis contrasting the response to active condition with the response to the control condition at each channel separately identified effects at Ch. 1, Ch. 2 and Ch. 3 at an uncorrected level, but only Ch. 1 remained significant after correction for multiple comparisons (see Table 8, Figs. 9 a and 10). Effect size and evidentiary strength were largest for Ch. 1 (*d* = 0. 62, BF_10_ = 12.9).

#### 4.2.2. Functionally-defined channel(s) of interest analysis

Defining a single channel of interest by contrasting the functional response to the active condition with the response to the control condition yielded statistically significant results for all fCOI approaches (see Table 9, Figs. 9 b–e and 10). However, effect sizes were nearly double (*d* = 1.03–1.1) and evidentiary strength was well over an order of magnitude greater (BF_10_ = 224–588) for within-subject fCOI approaches compared to those values obtained from results of between-subject fCOI method (*d* = 0.57, BF_10_ = 6.12) or the strongest results from the fixed-array analysis (*d* = 0.62, BF_10_ = 12.9).

Although all within-subjects fCOI methods proved highly sensitive to the effect in this synthetic dataset, evidentiary strength was about double for the leave-one-run-out method (BF_10_ = 588) compared to the other within-subject fCOI methods (BF_10_ = 224–235). The split-half and iterative contrasts yielded similar levels of sensitivity.

Effect sizes and evidentiary strength were slightly reduced (but remained strong) by increasing number of channels in the fCOI definition from a single channel of interest to two for all the within-subjects fCOI methods (see Table 9). Increasing the fCOI definition to include three channels substantially decreased effect sizes and evidentiary value for within-subjects fCOI (Table 9). This reduction was to be expected if results reflect ground truth, as a HRF was only inserted into the active condition for two channels per participant. Anatomical restriction slightly improved results for the within-subjects fCOI methods and had no effect on the results from the between-subjects fCOI method (see Table 10).

Together these results reinforce several of the main findings of the first two studies, that fCOI methods are broadly more sensitive than fixed-array methods and that within-subjects fCOI methods are more sensitive than the between-subjects method. All within-subject fCOI methods were highly sensitive to effect in this synthetic dataset, with the by-run method performing best. Increasing the number of channels or anatomically restricting fCOI search did not substantially improve sensitivity, but this is likely due to the fact that the synthetic response was already highly localized by design in this synthetic dataset.

##### 4.2.2.1. Consistency in channel selection.

As far as ground truth, a synthetic HRF was added to 2 of 3 target channels (Ch. 1, Ch. 2, Ch. 3) across this synthetic dataset. The two active channels were consistent within a single subject (100% with-subject consistency) but varied randomly between these three channels between subjects. Although the signal was distributed fairly evenly across the three target channels in the actual simulated data, Ch. 1 (37.5%) turned out to be the most frequent channel to receive signal, followed by Ch. 2 (32.5%), and then Ch. 3(30.0%).

All methods overwhelmingly identified the ground truth target channels as most relevant and rarely identified other channels. Specifically, all fCOI methods were consistent in identifying Ch. 1 as the most frequently selected channel for single channel definitions (by-subject = 90%, by-run = 39.1%, split-half = 42.5%, contrasts = 42.5%, see Fig. 9), Ch. 1 and Ch. 2 most frequently for two channel definitions (by-subject = 97.50%, by-run = 68.91%, split-half = 62.50%, contrasts = 62.50%), and Ch. 1, Ch. 2, and Ch. 3 most frequently for three channel definitions (by-subject = 100%, by-run = 71.37%, split-half = 66.67%, contrasts = 67.08%). This was consistent with ground truth regarding the localization of the signal, as the HRF was placed in only Ch. 1–3. Also as expected, between-subjects consistency decreased when the COI(s) were defined within-subjects since ground truth regarding the location of the signal varied between participants and within-subject fCOI methods allow for flexibility based on individual subject functional data.

Within-subject methods of COI also allowed for a calculation of within-subject consistency in channel selection. Although not perfect, metrics of within-subject consistency were reasonably high for all the fCOI analysis methods tested. Also consistent with ground truth that signal was inserted into two channels per participant, two channel definitions produced the highest levels of within-subject consistency for all methods and within-subject consistency dropped with three channel definitions. Specifically, the highest within-subject consistency was seen for the leave-one-run-out procedure, where the same channel was identified on average 93.13% of runs, the same two channels in 98% of runs when two channels of interests were used, and the same three channels on 90.77% of runs when three channels of interests were used as the COI definition. For the within-subjects split-half procedure, the same channel was identified for the same subject in both halves of the data 60% of the time for single channels fCOIs, the same two channels 82% of the time for two channel fCOI definitions, and the same channels 61.67% of the time when three channels of interests were used as the COI definition. For within-subjects iterative contrasts procedure, the same channel was identified for the same subject 75% of the iterations for single channel definitions, the same channels 91.25% of the time for two channel definitions, and 80.83% consistency was obtained with three channel definitions.

In sum, the results from the consistency analysis of the synthetic infant fNIRS dataset further validate that the fCOI methodologies employed are consistent with ground truth, even with added noise reasonably consistent with infant artifacts and physiology.

## 5. General discussion

Here we compared methods and choices in infant fNIRS data analysis in three studies, two studies using real infant fNIRS datasets and one study using a synthetic infant fNIRS dataset. For all of these datasets, we compared a traditional, fixed-array analysis with several functional channel of interest analysis approaches. FCOI methods differed in how they used functional data to identify channels of interest. We also tested whether including multiple channels in the fCOI or anatomically restricting the fCOI search changed the results. Most broadly, we found reasonably high correspondences between all methods in the actual channels identified as most responsive in each real dataset study and good correspondence between fCOI definitions and ground truth in the synthetic dataset. This suggests that despite some differences in sensitivity to the effects of interest between approaches, analytical choices, and study contexts, all were localizing the basic effects.

### 5.1. Fixed-array versus fCOI approaches

Across all studies, we found robust evidence that fCOI approaches are more sensitive than fixed-array analyses. These findings accord with the results and conclusions of Powell and colleagues (Powell et al., 2018), who also found that defining a channel of interest based on functional data proved a more sensitive method of analysis compared to a traditional fixed-array analysis of the same dataset. Furthermore, we report novel findings that within-subjects fCOI methods are generally more sensitive than a between-subjects fCOI method. This is likely because within-subjects methods can better account for individual subject variation in probe placement, scalp shape, and head circumference between participants, as they do not assume each channel measures from the same brain region in every participant as other methods do. Given this flexibility, fCOI analysis approaches may also be a good choice for combining data from subjects who have used different sorts of head probes (e.g., our Study 2 results).

One important qualification to these conclusions is that traditional fixed-array analyses or between-subjects fCOI approaches may allow more participants to be retained, since they do not require multiple trials/runs per subject to be implemented. Furthermore, with more subjects (here we had about 50% more) these methods showed sensitivity comparable to within-subjects fCOI methods. Thus, these methods may be preferable to within-subjects fCOI approaches when only a single trial per condition or single runs of good data are likely to be obtained for a substantial number of subjects or if it would be very costly to reject subjects with less than two runs/trials per condition due to the special or resource-intense nature of the study (e.g., clinical or intense longitudinal study).

Another important qualification is that some spatial resolution may be lost with the application of fCOI approaches compared to fixed-array approaches. As the channels being analyzed are allowed to flexibly vary between participants, the ability to attribute a response to a particular scalp channel, and, by inference a particular brain region, may be diminished. Furthermore, this problem is exacerbated as the search space of potential fCOI channels widens. This limit may be reduced by a priori anatomical restriction in the potential fCOI channels, a procedure our results show is not likely to diminish sensitivity. Nevertheless, fixed-array analyses or the addition of anatomical measures to relax the assumption of the fixed channel-space (i.e., subject-specific MRI, subject-specific scalp-probe alignment, Emberson et al., 2015, 2017; Jaffe-Dax et al., 2020) may be preferred to fCOI approaches if the research question involves identifying and differentiating particular brain regions from one another or other more anatomically-focused purposes.

### 5.2. A comparison of within-subject fCOI approaches

We also compared three within-subjects methods for defining the fCOI. When defining COIs based on functional data, one must decide on which data to use to define the COI and which data to use to test your effects, ensuring the definitions are made on data that is considered independent from test data (Vul and Kanwisher, 2010). In functional neuroimaging literature more broadly, this is often carried out by doing two distinct tasks, a localizer task and the experimental task (Poldrack, 2007; Saxe et al., 2006), or by defining the regions of interest and testing the effects on different subsets of data from the same task (Powell et al., 2018). Conducting two distinct tasks is often not practical or even possible in infant fNIRS studies, so we compared methods that use independent sub-setting of the data to define fCOIs and test for effects.

There were some differences in the top performing within-subjects fCOI methods between studies. For Study 1, we found that the leave-one-run-out and split-half methods yielded stronger effects than the iterative contrasts method. For Study 2, we found that the iterative contrasts method and the split-half method yielded stronger results than the leave-one-run-out method. In Study 3 we found that the leave-one-run-out produced the strongest effects. Although less sensitive relative to the by-run method, the split-half method and iterative contrasts methods also produced relatively strong results in Study 3. Across all studies, the largest effect sizes and evidentiary strength values were seen for the leave-one-run-out method. The split-half method, on the other hand, appeared to produce the most stable sensitivity across studies, resulting in statistical significance and completive metrics of sensitivity across all studies.

One difference between studies that may explain differences in top method that emerged may be the amount of data per subject. Study 1 and Study 3 included about double the data per subject as Study 2. Specifically, Study 2 subjects retained an average of 2.4 trials per condition, while those in Study 1 retained 4.5 and simulated subjects in Study 3 retained 7.17 trials per condition. At 2 trials per condition, the leave-one-run-out procedure is mathematically equivalent to the split-half method and may explain why it did not turn out as the top model in Study 2. In contrast, the leave-one-run-out procedure was the top method in the studies with more data per subject and its sensitivity appeared to increase as the number of trials per condition per subject increased. Thus, it may be the case that the leave-one-run-out method may be more effective when there are relatively more useable data points per condition for each participant (above 2), while the split-half and iterative contrasts methods may be better choices if the amount of data per subject is less. Future work should examine this issue more closely by varying the number of trials included in the leave-one-run-out method (and possibly other methods) to see how trial numbers retained for analysis differentially influence metrics of sensitivity.

### 5.3. Effects of number of channels included in fCOI

One analytical choice under investigation was whether including multiple channels in an fCOI definition changes the results. In our analysis, we directly compared a single channel definition to multiple channel definitions (2 or 3). Although results differed slightly depending on method and specific study, most effects either remained stable or improved slightly with multiple channel fCOI definitions compared to single channel fCOI definitions. There was both a priori reasons (Pitcher et al., 2011; Powell et al., 2018) and empirical results to think that dynamic face-sensitivity tested in Study 1 was distributed across multiple sub-regions within the posterior temporal lobe and, thus, multiple data channels. For both Studies 1 & 2, metrics of fCOI consistency suggested variability in the particular COIs selected across different subsets of the data for the same subjects. For Study 3 where the HRF was known to be localized to two channels, we saw the best performance with 2 channel fCOI models relative to single-channel or three-channels fCOI models. Our recommendation, then, based on these findings is that multiple channel fCOI definitions may want to be used in cases where the anticipated response is expected to be more distributed or other data-driven metrics from the dataset suggest multiple responsive channels. However, if this decision cannot be made and justified before data analysis begins, one should probably default to single channel fCOI definitions in order to avoid unnecessary researcher degrees-of-freedom in the analysis (Gelman and Loken, 2013).

### 5.4. Effects of anatomical restriction in fCOI

Yet another analytical choice is whether to anatomically restrict the COI search to a particular part of the head probe. Anatomically constrained analyses are common in the infant fNIRS literature because probes often encompass more of the head or scalp than required to identify a particular brain region of interest. This is done, in part, because spatial localization of the brain from the scalp is coarse and placements vary slightly between participants. Relatedly given the spatial resolution of fNIRS, even single channels are likely to encompass multiple brain regions. Anatomical restriction is also used because available fNIRS infant head probes are often modular or not easily reduced to fewer channels, even if the particular study calls for it. As a result, researchers often cover more scalp/brain than necessary to target the brain region(s) of interest in the study. Overall, we saw that restricting the fCOI search anatomically seemed to either improve or not have major effects on sensitivity as indicated by the metrics of effect size. On average, effect sizes remained similar when anatomical restriction was imposed in Study 1 and Study 3, and improved when anatomical restriction was imposed in Study 2.

An important qualification to this is that scalp coverage in our studies was already relatively focused. Other fNIRS systems or studies have broader head coverage, a trend that is likely to increase in the future with advancements in headgear technology. Anatomical restrictions may be more obvious as systems approach full or near full head coverage. A point for future work should be to determine the utility of single versus multiple channel fCOI definitions and anatomical restriction as scalp coverage varies. Different results and conclusions would likely be obtained in studies with broader head coverage, especially if coverage included distinct regions that were clearly not of interest to researchers in a particular study. Based on our observations here, though, we recommend that anatomical restriction be applied if there is good reason to think that some channels are irrelevant, either because of the modularity in equipment or because of a localization estimates from scalp positions (or other methods of sensitivity analysis). It may also be desirable if the research question demands localization or differentiation between activity in particular brain regions, an aspect of the recordings that can be limited by including more channels in the fCOI search space. However, like the case of number of channels of interest, if this decision cannot be made before data analysis, one should probably default to include all channels in the fCOI search space estimates in order to avoid unnecessary researcher degrees-of-freedom in the analysis (Gelman and Loken, 2013).

### 5.5. Broader limitations

There are several other limitations to be taken into account when considering our comparison of infant fCOI data analysis approaches. First, a single fCOI cannot be clearly equated to a single brain region in infants since a single channel measures several centimeters of cortical surface. As such, there could be multiple different functional responses over the extent of cortical surface even in a single channel measurement. Second, fCOI analyses require multiple trials per condition, in order to make COI definitions that are independent of the test data. The amount of data on which to make independent COI definitions and test is often limited with infants and, therefore, fCOI definitions within individuals may not always be possible or advisable as mentioned above. Third, the fCOI approach may not be suitable for studies where a particular functional response signature is not expected a priori or where expected responses are more highly distributed across the cortical surface. Fourth, special considerations should be given when retaining every subject is of key importance to the study and the expected data yielded from each subject may sometimes be a single acceptable run or a single acceptable trial per condition.

## 6. Summary

In summary, much of the previously published literature and current analysis approaches accept the assumptions of the fixed-array analysis approach that: (a.) if an infant fNIRS head probe is placed similarly and systematically across participants, then the scalp-brain correspondences are similar enough across participants that a given channel can be said to be targeting the same brain region across participants, and (b.) that head size either does not need to be accounted for as a factor or that it can be accounted for by some additional placement procedures or rules (e.g., place all probes relative to relative scalp landmark). Given what we know about scalp-brain correspondences, variance in head probe placement, and the exaggerated variance in localization that results from these issues in infants (Emberson et al., 2017; Lloyd-Fox et al., 2014), these assumptions are not strictly true for infants, even if we are willing to accept them in our work (or have been willing in the past). Methods that relax this fixed channel-space assumption are emerging. One such approach investigated here, the fCOI analysis approach, relaxes some of these assumptions, as a channel is considered relative and aligned between-subjects based on the functional response, instead of solely on its anatomical placement on the scalp. Here we report new empirical evidence that fCOI approaches are broadly more sensitive than traditional fixed-array approaches and within-subject fCOI approaches are more sensitive than between-subjects fCOI approaches when analyzing infant fNIRS data. Our findings both replicate and extend initial findings in the field (Powell et al., 2018). We further provide novel quantitative comparisons of several major analytic choices, revealing how different methodological choices affect sensitivity to provide an empirical basis on which researchers can make choices about their analyses in the future. In sum, fCOI approaches appear to be a powerful method for infant fNIRS data analysis that are flexible to individual differences in brain organization and methodology.

## Figures and Tables

**Table 1:** Statistical results of face-selectivity using a channel-by-channel fixed-array analysis.

Channel	t(19)	*P*	Cohen’s *d*	BF_10_
1	0.7118	.2426	0.1592	0.435
2	−0.2985	.6157	−0.0668	0.189
3	1.6751	.0552	0.3746	1.430
4	−0.3740	.6437	−0.0836	0.180
5	1.1186	.1386	0.2501	0.684
6	−1.6776	.9451	−0.3751	0.098
7	1.3275	.1000	0.2968	0.885
8	−0.5419	.7029	−0.1212	0.162
9	−1.2316	.8834	−0.2754	0.116
10	−0.6140	.7266	−0.1409	0.159

**Table 2:** Statistical results of face-selectivity using fCOI analysis approaches.

# of channels	Between Ss	Within Ss, Leave-one-run-out	Within Ss, Split-half	Within Ss, Iterative Contrasts
1	t(19) = 1.6751, *p* = .0552, *d* = 0.3746, BF_10_ = 1.42	t(19) = 2.2967, *p* = .0166*, *d* = 0.5136, BF_10_ = 3.76	t(19) = 1.7445, *p* = .0486*, *d* = 0.3901, BF_10_ = 1.58	t(19) = 1.0800, *p* = .1468, *d* = 0.2415, BF_10_ = 0.653
2	t(19) = 1.2179, *p* = .1191, *d* = 0.2723, BF_10_ = 0.772	t(19) = 1.9737, *p* = .0316*, *d* = 0.4413, BF_10_ = 2.23	t(19) = 2.0004, *p* = .0300*, *d* = 0.4473, BF_10_ = 2.32	t(19) = 1.2253, *p* = .1177, *d* = 0.2740, BF_10_ = 0.778
3	t(19) = 0.6713, *p* = .2550, *d* = 0.1501, BF_10_ = 0.417	t(19) = 1.7775 *p* = .0457*, *d* = 0.3975, BF_10_ = 1.66	t(19) = 2.0035 *p* = .0298*, *d* = 0.4480, BF_10_ = 2.34	t(19) = 1.0320, *p* = .1575, *d* = 0.2308, BF_10_ = 0.617

The * denotes statistical significance.

**Table 3:** Statistical results of face-selectivity applying anatomical restriction to fCOI approaches.

# of channels	Between-Ss	Within-Ss, leave-one- run-out	Within-Ss, split-half	Within-Ss, iterative contrasts
1	t(19) = 1.6751, *p* = .0552, *d* = 0.3746, BF_10_ = 1.42	t(19) = 1.8437, *p* = .0404*, *d* = 0.4123, BF_10_ = 1.83	t(19) = 2.6250, *p* = .0083*, *d* = 0.5870, BF_10_ = 6.64	t(19) = 1.0677, *p* = .1495, *d* = 0.2387, BF_10_ = 0.643
2	t(19) = 1.2179, *p* = .1191, *d* = 0.2723, BF_10_ = 0.772	t(19) = 1.3294, *p* = .0997, *d* = 0.2973, BF_10_ = 0.888	t(19) = 2.6703, *p* = .0076*, *d* = 0.5971, BF_10_ = 7.20	t(19) = 1.0605, *p* = .1511, *d* = 0.2371, BF_10_ = 0.638
3	t(19) = 1.3641, *p* = .0942, *d* = 0.3050, BF_10_ = 0.929	t(19) = 1.3158 *p* = .1020, *d* = 0.2942, BF_10_ = 0.873	t(19) = 2.4099 *p* = .0131*, *d* = 0.5389, BF_10_ = 4.56	t(19) = 1.0366, *p* = .1565, *d* = 0.2318, BF_10_ = 0.620

The * denotes statistical significance.

**Table 4:** Statistical results of sensitivity using a channel-by-channel fixed-array analysis with full dataset (*N* = 50).

Channel	*t*(df)	*p*	Cohen’s *d*	BF_10_
1	2.4842(48)	0.0083*	0.3549	4.88
2	3.2789(47)	0.0010*	0.4733	31.6
3	3.8259(49)	0.0002*	0.5411	140
4	−0.4267(48)	0.6642	−0.0610	0.115
5	0.5945(49)	0.2774	0.0841	0.261
6	1.3874(49)	0.0858	0.1962	0.687

The * denotes statistical significance after correction for multiple comparisons.

**Table 5:** Statistical results of sensitivity using a channel-by-channel fixed-array analysis with only participants that contributed at least two complete runs (*N* = 34).

Channel	*t*(33)	*p*	Cohen’s *d*	BF_10_
1	2.1063	0.0214	0.3612	2.51
2	2.2892	0.0143	0.3926	3.52
3	2.4298	0.0104	0.4167	4.62
4	0.9242	0.1809	0.1586	0.442
5	0.3636	0.3592	0.0624	0.249
6	1.7469	0.0450	0.2996	1.37

The * denotes statistical significance after correction for multiple comparisons.

**Table 6:** Statistical results for belief processing using fCOI analysis approaches.

# of channels	Between-Ss	Within-Ss, leave-one-run-out	Within-Ss, split-half	Within-Ss, iterative contrasts
1	t(33) = 0.9320, *p* = .1790, *d* = 0.1598, BF_10_ = 0.446	t(33) = 1.5369, *p* = .0669, *d* = 0.2636, BF_10_ = 0.990	t(33) = 1.8877, *p* = .0339*, *d* = 0.3237, BF_10_ = 1.72	t(33) = 2.0284, *p* = .0253*, *d* = 0.3479, BF_10_ = 2.19
2	t(33) = 2.4623, *p* = .0096*, *d* = 0.4223, BF_10_ = 4.93	t(33) = 1.6191, *p* = .0575, *d* = 0.2777, BF_10_ = 1.12	t(33) = 1.9394, *p* = .0305*, *d* = 0.3326, BF_10_ = 1.88	t(33) = 1.9174, *p* = .0319*, *d* = 0.3288, BF_10_ = 1.81
3	t(33)=2.0421, *p* = .0246*, *d* = 0.3502, BF_10_ = 2.24	t(33) = 1.8330, *p* = .0379*, *d* = 0.3144, BF_10_ = 1.57	t(33) = 2.1324, *p* = .0203*, *d* = 0.3657, BF_10_ = 2.64	t(33) = 2.2223, *p* = .0166*, *d* = 0.3811, BF_10_ = 3.11

The * denotes statistical significance.

**Table 7:** Statistical results for belief processing using fCOI analysis approaches with anatomical restriction.

# of channels	Between-Ss	Within-Ss, leave-one- run-out	Within-Ss, split-half	Within-Ss, iterative contrasts
1	t(33) = 0.9320, *p* = .1790, *d* = 0.1598, BF_10_ = 0.446	t(33) = 3.7617, *p <*.001*, *d* = 0.6451, BF_10_ = 92.8	t(33) = 3.8045, *p <*.001*, *d* = 0.6525, BF_10_ = 103	t(33) = 3.4694, *p <*.001*, *d* = 0.5950, BF_10_ = 45.4
2	t(33) = 2.4623, *p* = .0096*, *d* = 0.4223, BF_10_ = 4.93	t(33) = 3.2810, *p* = .0012*, *d* = 0.5627, BF_10_ = 29.1	t(33) = 3.1847, *p* = .0016*, *d* = 0.5462, BF_10_ = 23.2	t(33) = 2.9803, *p* = .0027*, *d* = 0.5111, BF_10_ = 14.6

The * denotes statistical significance.

**Table 8:** Statistical results from synthetic fNIRS dataset using a channel-by-channel fixed-array analysis.

Channel	t(19)	*p*	Cohen’s *d*	BF_10_
1	2.9889	0.0038*	0.6683	12.9
2	2.8172	0.0055	0.6299	9.39
3	2.2173	0.0195	0.4958	3.30
4	−0.2161	0.5844	−0.0483	0.199
5	−0.3185	0.6232	−0.0712	0.186
6	0.1308	0.4486	0.0293	0.257
7	−0.1572	0.5616	−0.0351	0.207
8	−0.3299	0.6275	−0.0738	0.185
9	−0.7596	0.7716	−0.1699	0.144
10	−0.8037	0.7842	−0.1797	0.141

**Table 9:** Statistical results from synthetic fNIRS dataset using fCOI analysis approaches.

# of channels	Between-Ss	Within-Ss, leave-one-run-out	Within-Ss, split-half	Within-Ss, iterative contrasts
1	t(19) = 2.5794, *p* = .0092*, *d* = 0.5768, BF_10_ = 6.12	t(19) = 4.9249, *p* = .000047*, *d* = 1.1013, BF_10_ = 588	t(19) = 4.6987, *p* = .000078*, *d* = 1.0507, BF_10_ = 235	t(19) = 4.6259, *p* = .000092*, *d* = 1.0344, BF_10_ = 224
2	t(19) = 3.0193, *p* = .0035*, *d* = 0.6751, BF_10_ = 13.7	t(19) = 4.5276, *p* = .00015*, *d* = 1.0124, BF_10_ = 265	t(19) = 4.2907, *p* = .00019*, *d* = 0.9594, BF_10_ = 114	t(19) = 4.3585, *p* = .00016*, *d* = 0.9746, BF_10_ = 161
3	t(19) = 3.1301, *p* = .0029*, *d* = 0.6999, BF_10_ = 16.8	t(19) = 3.1078 *p* = .0029*, *d* = 0.6949, BF_10_ = 16.1	t(19) = 2.8369 *p* = .0053*, *d* = 0.6343, BF_10_ = 9.75	t(19) = 2.9041, *p* = .0045*, *d* = 0.6494, BF_10_ = 10.8

The * denotes statistical significance.

**Table 10:** Statistical results from synthetic fNIRS dataset using fCOI analysis approaches with anatomical restriction.

# of channels	Between-Ss	Within-Ss, leave-one-run-out	Within-Ss, split-half	Within-Ss, iterative contrasts
1	t(19) = 2.5794, *p* = .0092*, *d* = 0.5768, BF_10_ = 6.12	t(19) = 4.9873, *p* = .000041*, *d* = 1.1152, BF_10_ = 666	t(19) = 4.7666, *p* = .000067*, *d* = 1.0658, BF_10_ = 270	t(19) = 4.6622, *p* = .000084*, *d* = 1.0425, BF_10_ = 241
2	t(19) = 3.0193, *p* = .0035*, *d* = 0.6751, BF_10_ = 13.7	t(19) = 4.5898, *p* = .00010*, *d* = 1.0263, BF_10_ = 301	t(19) = 4.6268, *p* = .000091*, *d* = 1.0346, BF_10_ = 254	t(19) = 4.5764, *p* = .00010*, *d* = 1.0233, BF_10_ = 233
3	t(19) = 3.1301, *p* = .0028*, *d* = 0.6999, BF_10_ = 16.8	t(19) = 3.2412, *p* = .0021*, *d* = 0.7248, BF_10_ = 20.8	t(19) = 3.1741, *p* = .0025*, *d* = 0.7098, BF_10_ = 16.7	t(19) = 3.2249, *p* = .0022*, *d* = 0.7211, BF_10_ = 17.5

The * denotes statistical significance.

## Data Availability

Sample analytic code that forms the basis for all analyses reported here, as well as a simulated infant fNIRS dataset (i.e., study 3 dataset) are publicly available on Open Science Framework (https://osf.io/mx49d/). Other study materials and code related to the results reported here are available from the authors upon reasonable request.

## Footnotes

1 There are many ways to approach independently identifying regions of interest. The most straightforward would be to collect two full sets of data on every participant and use one set to define the ROI and then use that ROI definition to test the hypotheses in the second set. However, it is important to note that the amount of data that one can successfully acquire in infants is limited compared to a typical study with adults or older children. As such, it is not often feasible to collect two full sets of data or collect data on both a localizer task and the main task.

2 It is important to note that conducting the between-subject fCOI analysis, a method that does not require multiple runs per subject, on the full sample (N = 50), also yielded a statistically significant effect (*t* (48) = 3.5249, p <.001) and medium effect size (*d* = 0.50) comparable to that observed for the fixed-array method.

## Abbreviations:

fCOI: functional channel of interest
fNIRS: functional near-infrared spectroscopy
